# Novel mechanism for OSM-promoted extracellular matrix remodeling in breast cancer: LOXL2 upregulation and subsequent ECM alignment

**DOI:** 10.1186/s13058-021-01430-x

**Published:** 2021-05-19

**Authors:** Simion C. Dinca, Daniel Greiner, Keren Weidenfeld, Laura Bond, Dalit Barkan, Cheryl L. Jorcyk

**Affiliations:** 1 Biomolecular Sciences Graduate Program, Boise State University, 1910 University Drive, MS1515, Boise, ID 83725 USA; 2 Department of Biochemistry, University of Utah School of Medicine, Salt Lake City, UT 84112 USA; 3 Department of Biological Sciences, Boise State University, 1910 University Drive, MS1515, Boise, ID 83725 USA; 4 Department of Human Biology and Medical Sciences, University of Haifa, Haifa, Israel; 5 Biomolecular Research Center, Boise State University, 1910 University Drive, MS1515, Boise, ID 83725 USA

**Keywords:** Cytokines, OSM, Inflammation, LOXL2, Extracellular matrix, Collagen, Breast cancer, Tumor microenvironment, IL-6, Metastasis

## Abstract

**Background:**

Invasive ductal carcinoma (IDC) is a serious problem for patients as it metastasizes, decreasing 5-year patient survival from > 95 to ~ 27%. The breast tumor microenvironment (TME) is often saturated with proinflammatory cytokines, such as oncostatin M (OSM), which promote epithelial-to-mesenchymal transitions (EMT) in IDC and increased metastasis. The extracellular matrix (ECM) also plays an important role in promoting invasive and metastatic potential of IDC. Specifically, the reorganization and alignment of collagen fibers in stromal ECM leads to directed tumor cell motility, which promotes metastasis. Lysyl oxidase like-2 (LOXL2) catalyzes ECM remodeling by crosslinking of collagen I in the ECM. We propose a novel mechanism whereby OSM induces LOXL2 expression, mediating stromal ECM remodeling of the breast TME.

**Methods:**

Bioinformatics was utilized to determine survival and gene correlation in patients. IDC cell lines were treated with OSM (also IL-6, LIF, and IL-1β) and analyzed for LOXL2 expression by qRT-PCR and immunolabelling techniques. Collagen I contraction assays, 3D invasion assays, and confocal microscopy were performed with and without LOXL2 inhibition to determine the impact of OSM-induced LOXL2 on the ECM.

**Results:**

Our studies demonstrate that IDC patients with high LOXL2 and OSM co-expression had worse rates of metastasis-free survival than those with high levels of either, individually, and LOXL2 expression is positively correlated to OSM/OSM receptor (OSMR) expression in IDC patients. Furthermore, human IDC cells treated with OSM resulted in a significant increase in LOXL2 mRNA, which led to upregulated protein expression of secreted, glycosylated, and enzymatically active LOXL2. The expression of LOXL2 in IDC cells did not affect OSM-promoted EMT, and LOXL2 was localized to the cytoplasm and/or secreted. OSM-induced LOXL2 promoted an increase in ECM collagen I fiber crosslinking, which led to significant fiber alignment between cells and increased IDC cell invasion.

**Conclusions:**

Aligned collagen fibers in the ECM provide pathways for tumor cells to migrate more easily through the stroma to nearby vasculature and tissue. These results provide a new paradigm through which proinflammatory cytokine OSM promotes tumor progression. Understanding the nuances in IDC metastasis will lead to better potential therapeutics to combat against the possibility.

**Supplementary Information:**

The online version contains supplementary material available at 10.1186/s13058-021-01430-x.

## Background

Ductal carcinoma is the most commonly diagnosed form of breast cancer in women. It is classified as either pre-invasive ductal carcinoma in situ (DCIS) or invasive ductal carcinoma (IDC) [1]. If left undetected or untreated, IDC leads to tumor metastasis, which drops patient 5-year survival from > 95 to ~ 27% [2]. Due to the negative impact metastatic lesions have on patient survival, it is critical to understand the mechanisms that promote metastasis. IDC exists in an inflammatory microenvironment, saturated by cytokines released from tumor-infiltrating macrophages and neutrophils present in the stromal extracellular matrix (ECM) [3–5]. Interleukin-6 (IL-6)-related cytokines, such as oncostatin M (OSM), activate signaling pathways that stimulate the metastasis of IDC cells [6–9]. Identifying and exploiting novel mechanisms that increase invasive and metastatic potential of IDC is of paramount importance in creating therapeutics to disrupt metastasis.

The current paradigm in inflammation and cytokine-induced ductal tumor development is IL-6 signaling promotes progression and metastasis [10–12]. However, research shows that IL-6’s sister cytokine OSM also promotes invasion and metastasis in a manner independent of IL-6 [13–16]. Signaling is prompted when OSM binds to the gp130 receptor subunit, which leads to the recruitment and dimerization of OSM receptor β (OSMRβ) and the formation of the receptor complex (OSMR) [6–9]. Currently, it is thought that OSM promotes metastasis by stimulating an epithelial-to-mesenchymal transition (EMT) in breast ductal carcinoma cells through the up- or downregulation of specific genes that disturb cell polarity, promoting differentiation and motility [17–19]. EMT is stimulated through destabilized localization of E-cadherin or its downregulation, as well as an increase in Vimentin, Snail-1, and N-cadherin gene expression [20, 21]. Our lab has also demonstrated that OSM induces the upregulation of (i) vascular endothelial growth factor (VEGF) that leads to angiogenesis [22], (ii) circulating tumor cell (CTC) numbers [9], and (iii) lung and bone metastases in vivo [23]. Previous research highlights the important and multifaceted role that OSM signaling plays in IDC progression and metastasis. However, the impact OSM signaling has on ECM remodeling in the tumor microenvironment (TME), has yet to be explored.

The extracellular matrix (ECM) plays an integral role in tumor progression, as remodeling the ECM of the tumor microenvironment is critical for ductal carcinoma invasion and metastasis [24–27]. Invasive ductal carcinoma cells must degrade and break through a specialized ECM (basement membrane, composed primarily of collagen IV, BME) before migrating through the stroma to promote invasion to nearby tissues and vasculature [28–30]. Proinflammatory cytokines have previously been associated with promoting the expression of BME-degrading enzymes [31–33]. Once the BME is degraded, IDC cells modify the surrounding stromal ECM by secreting enzymes that remodel the structural proteins, present in stromal ECM, promoting invasion and metastasis [34–37]. Here our research suggests that OSM signaling plays a prominent role in IDC cell’s ability to remodel the primary constituent of stromal ECM, collagen I. Remodeling occurs as OSM induces the expression and secretion of the matrix remodeling enzyme lysyl oxidase like-2 (LOXL2) in IDC cells.

Similar to OSM, LOXL2 expression has been linked to a worse prognosis in IDC patients, and increased invasion and metastasis of breast tumor cells [38–43]. LOXL2 is part of a family of monoamine oxidases known as lysyl oxidases, this family includes lysyl oxidase (LOX) and LOXL1-4 [44, 45]. LOXL2 is copper-dependent enzyme containing a lysyl tyrosylquinone (LTQ) site in the active domain for turning peptidyl lysine and hydroxylysine into peptidyl allysine and hydroxyallysine on collagen and elastin [46]. These aldehydes spontaneously react to form a covalent bond between themselves, with hydrogen peroxide (H_2_O_2_) as a byproduct [47]. The covalent bonds formed in this reaction are collectively known as “crosslinking,” which leads to changes in ECM structure, density, and stiffness [48, 49]. LOXL2 is present in the cell cytoplasm before it is glycosylated at amino acids N593 and N627, and it promotes collagen I fiber alignment and crosslinking when secreted [50, 51]. Alignment of stromal collagen I fibers facilitate directed tumor cell motility towards nearby vasculature and/or tissue, as opposed to haphazard motility that occurs with random collagen I fiber alignment [52, 53]. Research also suggests that LOXL2 also has a cell autonomous role. LOXL2 was shown to promote an EMT of breast cancer cells resulting in invasive and stem-like properties of the cancer cells [43, 54–56].

There is currently a gap in knowledge regarding the role that proinflammatory cytokines play in ECM remodeling of the TME, specifically the surrounding stroma. Our studies demonstrate that OSM signaling promotes the expression and secretion of enzymatically active LOXL2. We also demonstrate that OSM-induced LOXL2 leads to significantly more crosslinking and alignment of ECM collagen I, the main constituent of the stroma. Further studies show that OSM-induced LOXL2 leads to increased IDC cell invasion in a 3D collagen matrix. Understanding how OSM regulates LOXL2 production, and more broadly matrix remodeling of the stroma, will shed light on the effect inflammation has on the TME of IDC patients. This is critical, as our research demonstrates that high OSM and LOXL2 co-expression in IDC patients leads to a drastic decrease in metastasis-free survival. Hence, our research will lead to a better understanding of the dynamic nature of inflammation promoted metastasis.

## Materials and methods

### Cells and cell culture

Human breast cancer cell lines used in experiments were purchased from the American Type Culture Collection (ATCC; Manassas, VA). Human luminal A MCF7 and T47D [ER+, PR+, HER2−] and triple negative basal B MDA-MB-231 [ER−, PR−, HER2− were cultured in RPMI 1640 (Genesee Scientific; San Diego, CA), while BT474 [ER+, PR+, HER2+] and triple negative basal A MDA-MB-468 [ER−, PR−, HER2−] cells were cultured in DMEM (Genesee Scientific). Sk-Br-3 [ER−, PR−, HER2+] breast cancer cells were cultured using McCoy’s 5A media (ATCC). All cell media contained 10% v/v Fetal Clone III (Thermo Fisher; Waltham, MA) and 1% v/v penicillin/streptomycin (Genesee Scientific). Cells were cultivated in tissue culture treated T-75 flasks (Genesee Scientific) kept in a Model 3110 (Forma Scientific; Marietta, OH) incubator at 37 °C and 5% CO_2_. Cells grown to ~ 75% confluence before plating for experiments. Cells were treated with recombinant human OSM, IL-6, leukemia inhibitory factor (LIF) (25 ng/mL), and/or interleukin 1β (IL-1β; 10 ng/mL) from Peprotech Inc. (Rocky Hill, NJ) at various time intervals depending on the experiment and highlighted in the figures.

### Gene correlation (RNA-Seq)

The Cancer Genome Atlas (TCGA) RSEM counts associated with breast invasive carcinoma (BRCA), glioblastoma multiforme (GBM), prostate adenocarcinoma (PRAD), and ovarian cancer (OV) were downloaded from the Broad GDAC Firehose repository (https://gdac.broadinstitute.org/). Using python, RSEM count data was standardized to *Z*-score for comparison and outlier patients above and below 3 standard deviations were removed from the dataset. Genes were then plotted, and correlation was assessed by Pearson coefficient using the SciPy package [57]. The line of best fit was determined by linear regression using the Polyfit function in the SciPy package. Specific code used for the analysis is available upon request at GitHub.

### Patient metastasis-free survival

The data associated with van de Vijver et al. [58] was downloaded and coded to ensure key results and figures from the data could be generated. Observed events were coded to be positive outcomes for metastasis, or identified as a death from cancer without metastasis, and a censored event to be any other outcome (by van de Vijver’s definitions). The survival function was censored at 10 years to reduce the influence of the few cases with far longer survival times. Survival plots were created with the Survminer (Kassambara et al., 2019) and Survival (Therneau, 2015) libraries in R. OSM and LOXL2 cut points were based on the Maximally Selected Rank Statistic [59], which algorithmically searches the data for optimal cut points.

### Real-time quantitative reverse transcription-polymerase chain reaction (qRT-PCR)

RNA extraction from treated cell cultures was performed using RNA STAT-60 (Tel-Test, Inc.; Friendswood, TX) following the standardized protocol on Tel-Test’s website. Isolated RNA concentration and quality was analyzed using a Nano-Dropper2000 (Thermo Scientific; Waltham, MA) and the agarose bleach gel protocol [60], respectively. Synthesis of cDNA was prepared using High-Capacity cDNA Reverse Transcription Kit (Applied Biosystems; Foster City, CA) and 1 μg of sample mRNA. Combining SYBR Green MasterMix (Bio-Rad; Hercules, CA) with sample cDNA, each sample was run in duplicate, at a minimum, on a 96-well plate. Roche Light Cycler 98 and accompanying software was used to determine mRNA expression.

**Table:** 

Oligonucleotide	Base pairs
*LOXL2* Forward	(5′)-AGGTATCGATGCCCATTCATGA-(3′)
*LOXL2* Reverse	(3′)-GGATCAACTGATAGCTGAATAC-(5′)
*GAPDH* Forward	(5′)-GTTAGCTAGGAATAGCGATAGA-(3′)
*GAPDH* Reverse	(3′)-AGCATTAGTACAGTTAGCATGC-(5′)

### Immunoblot assay

Cells were lysed using RIPA, 1% v/v Protease Inhibitor Cocktail (Sigma-Aldrich; St. Louis, MO), and 100× Halt™ Phosphatase Inhibitor (Thermo Fisher). Ten micrograms of total protein was loaded per well, as determined by Peirce™ BCA Protein Assay Kit (Thermo Fisher). The Chameleon® Duo Protein Ladder (LiCor Biosciences; Lincoln, NE) was used as a protein molecular weight marker. Proteins were separated using Tris-Glycine SDS-PAGE gels and transferred onto nitrocellulose membranes (Thermo Scientific). Subsequently, the membrane was thoroughly dried and rewetted with ddH2O. Five milliliters of REVERT™ Total Protein (LiCor Biosciences) stain was added before the REVERT wash solution. The rinsing and the blot was imaged using Odyssey CLx (LiCor Biosciences). The nitrocellulose membrane was blocked using Odyssey PBS Blocking Buffer (LiCor Biosciences) and incubated with the following primary antibodies in addition to 0.2% Tween20: LOX (1:500, Santa Cruz Biotech; Dallas, TX), LOXL1 (1:200, Santa Cruz Biotech), LOXL2 (1:1,000, Genetex; Irvine, CA), LOXL3 (1:200, Santa Cruz Biotech), LOXL4 (1:200, Santa Cruz Biotech), E-cadherin (1:500, Abcam; Cambridge, UK), Snail-1 (1:1,000, Cell Signaling Technology; Danvers, MA), and GAPDH (1:500, Santa Cruz Biotech). Membranes were further incubated using 800 channel fluorophore conjugated donkey secondary antibodies (1:15,000, LiCor Biosciences), or HRP conjugated secondary antibodies (1:10,000, Jackson ImmunoResearch Laboratories; West Grove, PA) prior to addition of ECL substrate (Thermo Fisher). The target proteins were then visualized using the Odyssey CLx and quantified using LiCor Image Studio software. Proteins were then normalized either against a REVERT total protein stain or GAPDH expression and compared against non-treated controls.

### De-glycosylation assay

MCF7 and MDA-MB-468 cells are treated with OSM for 24 h before samples were lysed with RIPA. One microgram of Rapid PNGase F (Cell Signaling) enzyme was added to 10 μg of total protein from OSM-treated cell lysates. The assay was performed following the accompanying Rapid PNGase F protocol. LOXL2 proteins were visualized using the immunoblot techniques described above.

### RNAi transfections

Using Qiagen’s protocol, the best LOXL2 knockdown in MCF7 cells came from the combination of 5 nM siLOXL2 #2 and 5 nM siLOXL2 #3 (Qiagen; Hilden, GER), called siLOXL2 (2/3), with 3 μL Transfection reagent (Qiagen) for 48 h. To make the control siControl, 5 nM of scrambled siRNA (Qaigen) with 3 μL transfection reagent was used. The optimal cell density for transfection was 125,000 MCF7 cells in a 6-well plate. The MCF7-shLOXL2 and MCF7-shCTRL cells used in this experiment have previously been published and characterized [43].

### Lysyl oxidase activity assay

MCF7 cells were transfected with siCTRL or siLOXL2 (2/3) for 48 h then treated with OSM in serum and phenol red-free RPMI 1640 for 24 h. The conditioned media (CM) was collected and immediately centrifuged at 8000*g* for 10 min to remove cellular debris. Lysates were collected to confirm LOXL2 knockdown. Conditioned media (1.75 mL) from each sample was added to separate 3 K filter tubes (MilliporeSigma; Burlington, MA), which were centrifuged at 4000*g* in a swinging bucket centrifuge for 30 min. The rest of the assay was formulated using the recipe previously published with volumes adjusted to fit within a 96-well fluorescence compatible plate (Thermo Fisher) [61]. The plates were read every 30 s using a BioTek Mx plate reader with Ex/Em 490/540 and 10 nm bandwidth.

### LOXL2 ELISA

The LOXL2 ELISA (R&D Systems; Minneapolis, MN) was performed and analyzed according to the protocol provided by the manufacturer. Protein was concentrated from CM using acetone precipitation [62]. For each condition, one-part CM was mixed with four parts of 100% acetone chilled to − 80 °C, and placed back into a − 80 °C freezer overnight. The CM was centrifuged using 13,000*g* for 10 min at − 10 °C. The acetone was decanted and the protein precipitates were dried for 20 min before being reconstituted with 1× dilution reagent (R&D Systems) containing 0.2% Tween20 at 1/3 the original volume. The samples were sonicated prior to addition to ELISA plate for a 3-fold increase in concentration.

### Immunofluorescence

Immunofluorescence staining was carried out as previously described [43]. Briefly, cells were cultured in 8-well chamber glass slides, fixed for 5 min with 4% PFA containing 5% sucrose and 0.1% Triton X-100, and re-fixed for an additional 25 min with 4% PFA containing 5% sucrose. The cells were washed 10 min with PBS and an additional 10 min with PBS containing 0.05% Tween 20. Fixed cells were blocked with IF buffer (130 mM NaCl, 7 mM Na_2_HPO_4_, 3.5 mM NaH_2_PO_4_, 7.7 mM NaN_3_, 0.1% BSA, 0.2% Triton X-100, 0.05% Tween 20) supplemented with 10% donkey serum for 1 h and incubated overnight at 4 °C with mouse monoclonal [HECD-1] to E-cadherin (1:500, Abcam). The cells were washed three times with PBS for 15 min each and incubated for 1 h with donkey anti-mouse conjugated to Alexa Fluor®647 (1:200, Molecular Probes; Eugene, OR), washed as above, and mounted with VECTASHIELD mounting medium with 4′, 6-diamidino-2-phenylindole (DAPI). For F-actin staining, cells were incubated overnight with Alexa Fluor®488 Phalloidin (1:40) (Molecular Probes), washed three times with PBS for 15 min each, and mounted with VECTASHIELD mounting medium with DAPI. Immunofluorescent images were captured by a Nikon A1R confocal microscope.

### Nuclear fraction assay

Nuclear/cytoplasmic fractionation assay was carried out as previously described [63]. Cells were washed twice with PBS, scraped, and collected on ice into 1.5-mL microcentrifuge tubes. Tubes were spun with table-top centrifuge, and supernatant was discarded. Fractionation was performed with 0.1% NP40 in PBS. Cell pellets were triturated 5× with ice-cold 0.1% NP40 in PBS (900 μl for 10 cm dish) using P1000 micropipette that was cut at its end. Aliquots of 300 μL of these samples were placed into fresh tubes (designated as “Total”). The remaining samples were centrifuged for 1 min 16,200*g* to pellet nuclei. Aliquots of 300 μL of the supernatant were collected into fresh tubes (designated as “Cyto”). In total, 100 μl of 4× Laemmli sample buffer was added immediately to *Total* and *Cyto* samples. Nuclei pellets were resuspended with ice-cold 0.1% NP40 in PBS (1 mL for 10 cm dish), re-pelleted, and resuspended with 180 μL of 1× Laemmli sample buffer (designated as “Nuc”). *Total* and *Cyto* samples were sonicated using microprobes at level 2, twice for 5 s.

### Collagen I contraction assay

Rat-tail collagen I (Corning; Corning, NY) was used to form a 1.5 mg/mL collagen I matrix in a 35-mm petri dish with a 14-mm imbedded coverslip. On ice, rat-tail collagen I and 10× RPMI 1640 media (Corning) was diluted 1:10 with 1× PBS and adjusted with 0.1 M NaOH to bring the final pH to 7.4. The MCF7 and MDA-MB-468 cells were seeded homogeneously in the matrix before adding 400 μL containing 100,000 cells to each petri dish-imbedded coverslip. The matrix solution was incubated 20 min at 37C and 5% CO_2_. Phenol red-free RPMI 1640 media (Thermo Fisher – Gibco) was added to each sample along with OSM and either 500 μM pan-LOX inhibitor β-aminopropionitrile βAPN (Thermo Fisher) or 200 nM LOXL2/3-specific small molecule inhibitor PXS-5120A (Pharmaxis; New South Wales, AUS) for 48 h. Images were processed, and the area of the matrix was analyzed using ImageJ area measurement tools [64, 65].

### Live cell imaging

Live cell imaging was performed using the Leica SP8 white light confocal microscope system with attached Peltier, which maintains cells at 37 °C and 5% CO_2_. In total, 100,000 MCF7 cells were seeded into the same rat-tail collagen matrix described above for the *Collagen I Contraction Assay*. The samples were exposed to recombinant human OSM and 500 μM βAPN for 36 h prior to imaging. Collagen I fibers were visualized using reflectance mode confocal imaging [66, 67]. Cells were stained with membrane intercalating fluorescent dye Cell Tracker™ Red (Life Technologies; Carlsbad, CA) for 1-h pre-imaging at a 1:1,000 dilution in serum/phenol-free RPMI 1640. Each image consisted of 15 μm z-stacks split into 44 sections that were taken with four frames stitched together in a 2 × 2 format at × 63 magnification using a water immersion objective.

### Collagen I fiber analysis

Live cell images of MCF7 cells in collagen I “pucks” were analyzed using CurveAlign4.0 software developed at the University of Wisconsin [68, 69]. Selected regions of interest (ROI) were analyzed—the areas between the seeded cells and radiating outward perpendicular to the MCF7 cells as illustrated in Supplemental Figure 4. ROIs were utilized because accurate whole image analysis was not possible due to the varying directions of collagen I fiber alignment. We used sum[(fiber dispersion coefficient) × (# of features (fibers) for each ROI)] / (total sampled features in image) to determine the average level of fiber dispersion for collagen I in each treatment group. For the fiber dispersion coefficient, 0 equals completely random fibers, 1 means all fibers are in alignment.

### 3-Dimensional invasion assay

Utilizing the Oris™ 3D Invasion Assay (Platypus Technologies; Madison, WI), 30,000 MCF7-GFP/Luc breast cancer cells (Genecopoeia; Rockville, MD) were seeded into the same 1.5 mg / mL rat-tail collagen I solution as described in the *Collagen I Contraction Assay* methods. The cells were exposed to 50 ng/mL β-estradiol (estrogen) (Sigma-Aldrich; St. Louis, MO) in order to promote invasion. In addition, MCF7-GFP/Luc cells were treated with OSM and either 500 μM of pan-LOX inhibitor β-aminopropionitrile (βAPN) or 200 nM of LOXL2/3-specific small molecule inhibitor PXS-5120A for 5 days, or 120 h. The rest of the experiment was performed according to the specifications of the Oris™ 3D Invasion Assay protocol that the assay includes. Images were taken at day 0, as a control, and day 5 utilizing an EVOS FL (Life Technologies; Carlsbad, CA) fluorescent microscope with a × 4 objective and GFP filter to detect MCF7 cells expressing GFP. ImageJ was used for image processing and cell counting.

### Statistical analysis

Statistical analysis was performed using Prism 8.0 software. All significant results were determined by various statistical methods including Student’s *t* test, One-way ANOVA, two-way ANOVA, and log rank test. Significance is denoted as n.s. (not significant), *p* > 0.05, *p < 0.05, **p < 0.01, ***p < 0.001, and ****p < 0.0001.

## Results

### Elevated OSM and LOXL2 co-expression is associated with a faster onset of metastasis

**Fig. 1:**
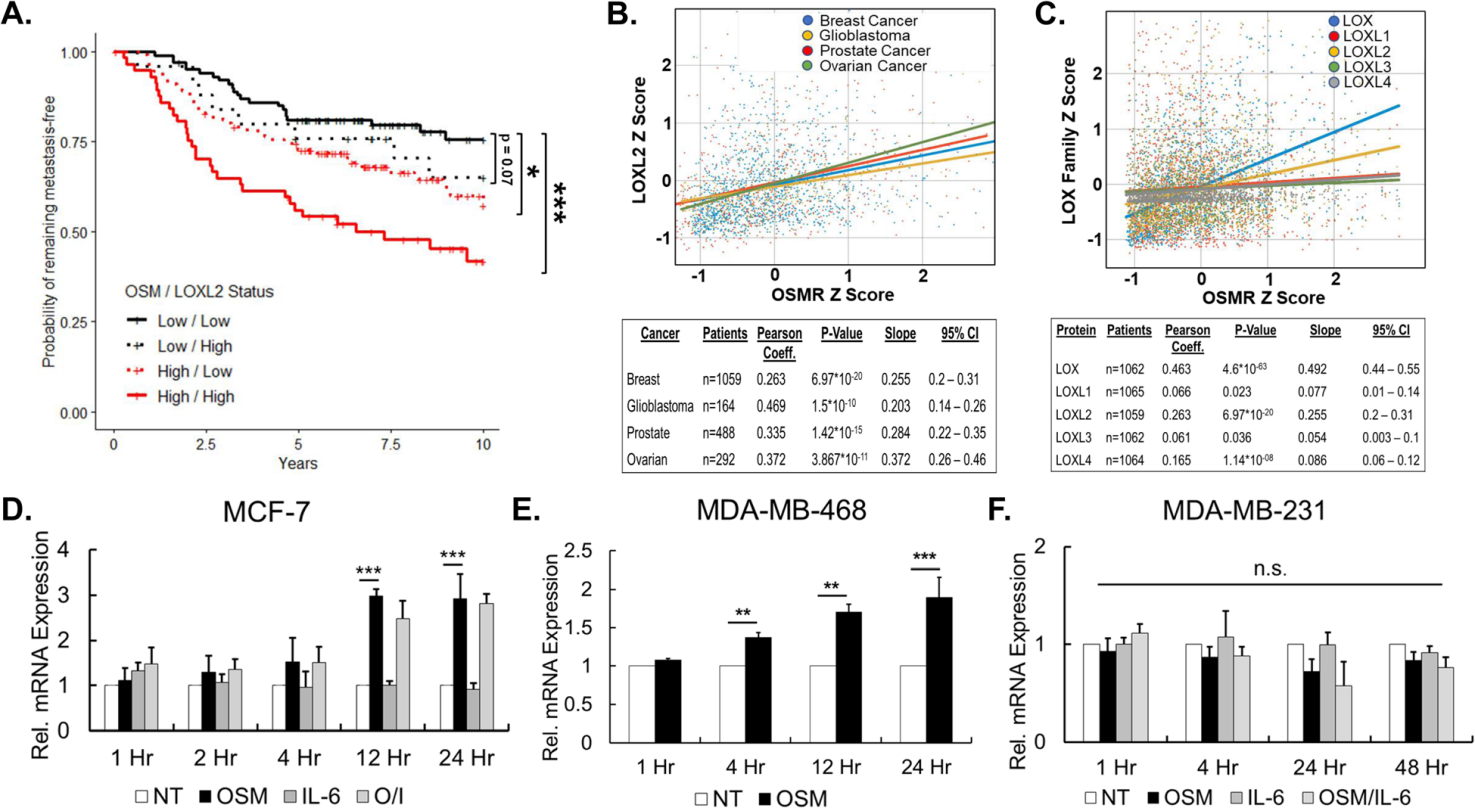
Co-expression of OSM and LOXL2 leads to drastically decreased metastasis-free survival. **a** Distant metastasis-free survival (DMFS) plotted from de Vijver et al. [58] invasive ductal carcinoma patient microarray database comparing low OSM/ low LOXL2 to low OSM/high LOXL2, high OSM/low LOXL2, and high OSM/high LOXL2 mRNA expression (*n* = 295). We observed a stronger negative impact on DMFS with high OSM and LOXL2 co-expression compared to high expression of OSM or LOXL2 separately (log rank test). **b** LOXL2 mRNA expression *Z*-score is positively correlated as measured by Pearson correlation coefficient to the beta subunit expression of OSM receptor (OSMR) mRNA expression *Z*-score in cancer patients analyzed from The Cancer Genome Atlas (TCGA) RNA-Seq database, specifically: breast cancer (BRCA), glioblastoma (GBM), prostate cancer (PRAD), and ovarian cancer (OV). Scatter plot consists of *Z*-score mRNA expression and line of best fit as determined by linear regression; a summary of the data is found in the accompanying table. **c** The mRNA *Z*-score of several LOXL2 family members exhibit positive Pearson correlation to OSMR mRNA *Z*-score in the breast invasive carcinoma dataset from TCGA. **d** qRT-PCR analysis of MCF7 luminal A invasive ductal carcinoma cells treated with OSM shows LOXL2 mRNA induction starting at 12 h and peaking at 24 h; there is no induction with IL-6. **e** qRT-PCR analysis of MDA-MB-468 basal A invasive ductal carcinoma cells treated with OSM also shows an increase in LOXL2 mRNA expression starting at 4 h. **f** qRT-PCR analysis of MDA-MB-231 basal B breast cancer cells, that constitutively express high levels of LOXL2, show no significant induction of LOXL2 mRNA expression by either OSM or IL-6 signaling. (All qRT-PCR experiments *n* = 3+; n.s. *p* > 0.05, **p < 0.01, ***p < 0.001; two-way ANOVA)

To determine whether high co-expression of LOXL2 and OSM mRNA is associated with increased rates of IDC metastasis in patients, we created a distant metastasis-free survival plot using microarray data from the van de Vijver et al. patient study consisting of 295 IDC patients [58]. This database was utilized because the patient population selected for this study and the metadata for metastasis is well characterized. We compared low OSM/ low LOXL2 to low OSM/ high LOXL2, high OSM/ low LOXL2, and high OSM/ high LOXL2 mRNA expression in patients and found that higher levels of OSM and LOXL2 mRNA combined, led to significantly more metastatic events in a 10-year span (Fig. 1a). High expression of each individual gene also led to faster onset of metastasis, but high OSM and high LOXL2 co-expression had a greater significant impact on distant metastasis-free survival.

Our lab, and others, have previously analyzed Oncomine™ and other breast cancer patient databases demonstrating a correlation between reduced recurrence-free survival (RFS) of breast cancer patients with higher expression of LOXL2 [38, 41, 43] and reduced survival rates with higher expression of OSM/OSMR [70, 71]. Taken together, these results confirm that high OSM and LOXL2 co-expression is associated with an overall worse prognosis in IDC patients than high expression of either gene alone.

### LOXL2 expression is positively correlated to OSM signaling through the OSMR in invasive ductal carcinoma patients

To assess the correlation between OSM signaling and LOXL2 expression in cancer patients, we analyzed the expression of LOXL2 mRNA in patients and compared it to OSMR mRNA expression. OSM is most often produced by neutrophils and macrophages found in the TME, and in turn, IDC cells, while having the capacity to secrete OSM, normally secrete none to very low levels [4, 5]. Therefore, comparing OSM mRNA expression from tumor samples directly against LOXL2 would not yield a highly relevant correlation. To assess OSM signaling, which occurs by OSM binding to the OSMR complex on tumor cells, we investigated the expression of the beta subunit of OSMR. Furthermore, increased levels of OSM in the TME promote the overexpression of OSMRβ mRNA in IDC cells [72–74]. To assess the correlation between OSMR and LOXL2, we used RNA-Seq data from The Cancer Genome Atlas (TCGA) to assay transcriptional expression of biopsied patient samples in several cancer subtypes. Specifically, LOXL2 was compared against OSMR expression in glioblastoma, breast cancer, prostate cancer, and ovarian cancer. We observed a moderate to weak, yet significant, positive Pearson correlation of 0.263 (*p* = 6.97 × 10^-20^) between OSMR expression and LOXL2 expression in breast cancer patients (Fig. 1b). There was also a weak to moderate positive, significant correlation between OSMR and LOXL2 expression in the other cancers investigated. To determine whether the correlation has a potential impact on gene expression we used a least squares linear regression to attain the line of best fit. Then we analyzed the slope and 95% confidence interval (CI) as illustrated in the table accompanying Fig. 1b, where a larger slope suggests a greater impact on gene expression. Each cancer analyzed had a positive slope above 0.2 and had a correlation between OSMR and LOXL2 mRNA expression. This data suggests that increasing OSMR mRNA is correlated with increasing LOXL2 transcripts in multiple forms of cancer; including breast cancer.

Next, we analyzed the breast cancer patient data for correlation between OSMR mRNA expression and other lysyl oxidase family members: LOX and LOXL1-4. We performed the same correlation analysis as above, but instead focused on OSMR and lysyl oxidase mRNA expression in breast cancer patients. We observed a significant, moderate to weak positive correlation (0.469 and 0.263) comparing OSMR expression to LOX and LOXL2 expression respectively, as determined by Pearson correlation analysis. While LOXL1/3/4 had significant Pearson correlation coefficients, the Pearson coefficients were considered very weak/negligible because they fall below 0.20 [75]. LOX and LOXL2 also generated a line of best fit with a positive slope above 0.2, when compared to OSMR expression, while the slopes for LOXL1/3/4 were approximately zero (Fig. 1c). These results suggest that increasing OSMR gene expression is correlated with increasing LOX and LOXL2 gene expression but not with LOXL1/3/4 expression. Further analysis of OSM mRNA expression in relation to lysyl oxidase mRNA expression again highlighted a significant correlation between OSM and all lysyl oxidases, except for LOXL4 (Supp. Figure 1). However, the Pearson correlation coefficient values for all the lysyl oxidases were below 0.25. The slopes for the lines of best fit for LOX and LOXL1-3 were also positive, but had large 95% CI. This data suggests that OSM gene expression is slightly correlated to increased LOX and LOXL1-3 expression. Taken together, these results confirm the positive correlation between OSM signaling and lysyl oxidase mRNA expression in breast cancer patients.

### OSM induces LOXL2 expression

As the human breast cancer patient data demonstrated a correlation between the proinflammatory cytokine OSM and the collagen crosslinking enzyme LOXL2, we set out to determine whether OSM could promote the expression of LOXL2 at the transcriptional level. qRT-PCR was performed on three IDC cell lines with varying estrogen receptor (ER), progesterone receptor (PR), and ErbB2 (HER2) status: luminal A MCF7 (ER+ PR+ HER2−), basal A MDA-MB-468 (ER− PR− HER2−), and basal B MDA-MB-231 (ER− PR− HER2−). These cell lines were chosen because they represent increasing tumor cell aggressiveness and invasiveness, respectively [76], and they each express receptors for OSM/IL-6 cytokines [22, 77]. The cells were treated with recombinant human OSM (25 ng/mL), IL-6 (25 ng/mL), or both for 1, 2, 4, 12, 24, and 48 h and compared against untreated controls. Our cells were treated with cytokine concentrations designed to saturate the IDC cells, to mimic an actual TME, where cytokines have been shown to be present in high concentrations due to secretion by tumor-associated neutrophils, macrophages, and fibroblasts [70, 78–80]. qRT-PCR analysis of MCF7 cells showed that OSM treatment induced a ~ 3-fold increase in LOXL2 mRNA, relative to the non-treated controls, at 12 and 24 h, whereas IL-6 treatment produced no significant change in LOXL2 mRNA (Fig. 1d). In MDA-MB-468 cells, OSM induced LOXL2 mRNA by ~ 2-fold at 24 h (Fig. 1e). In MDA-MB-231 cells, there was no significant change in expression of LOXL2 mRNA with any treatment groups (Fig. 1f). No effect on LOXL2 expression was somewhat expected since ER− MDA-MB-231 cells are already highly invasive, which can limit the impact of OSM signaling on promoting invasive potential [9]. MDA-MB-231 cells produce high levels of LOXL2, as highlighted in the following paragraph, limiting further induction by OSM. These results demonstrate that OSM signaling leads to an increase in LOXL2 mRNA expression in IDC cells.

**Fig. 2:**
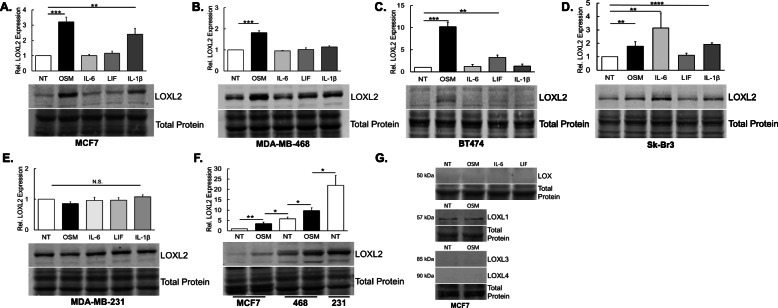
OSM promotes LOXL2 protein expression. All experiments and results pertain to immunoblot assays run with 10–20 μg total protein. **a** MCF7 breast cancer cells were treated with OSM, IL-6, LIF, and IL-1β for 24 h. Our analysis showed that only OSM and IL-1β promoted a significant upregulation of LOXL2 protein expression. **b** In analyzing MDA-MB-468 breast cancer cells, the same treatments that are described above were utilized. We observed that only cells treated with OSM had significantly induced LOXL2 protein expression. **c** BT474 breast cancer cells, under the same conditions, showed a significant increase in LOXL2 expression with OSM and LIF treatments. **d** In Sk-Br-3 breast cancer cells, we observed a significant increase in LOXL2 expression with OSM and IL-6 treatment. **e** We again used the same treatments in MDA-MB-231 breast cancer cells. LOXL2 expression was not significantly affected by either OSM, IL-6, LIF, or IL-1β treatment after 24 h. **f** Relative LOXL2 protein expression was compared among three breast cancer cell lines treated with OSM. From least invasive (MCF7) to the most (MDA-MB-231), we observed a stepwise increase in LOXL2 protein expression. OSM treatment bridges LOXL2 expression between cells. **g** MCF7 cells were treated for 24 h with OSM; OSM, IL-6, and LIF for LOX expression. No changes are observed in lysyl oxidase expression; LOXL1 is constitutively expressed. (All experiments *n* = 3+; n.s. *p* > 0.05, **p < 0.01, ***p < 0.001; Students *t* test)

To determine whether OSM-induced LOXL2 mRNA translates to the protein level, we performed immunoblot blot assays. Analysis was performed on MCF7, MDA-MB-231, MDA-MB-468, BT474 (ER+ PR+ HER2+), and Sk-Br-3 (ER− PR− HER2+) IDC cell lines treated with OSM, IL-6, LIF (all at 25 ng/mL), and IL-1β (10 ng/mL) for 24 h before cell lysates were collected and compared against untreated controls. Analysis of proinflammatory cytokines in the IL-6 family, as well as IL-1β, was performed to determine whether LOXL2 induction is unique to OSM signaling or has the potential to be broadly applicable to proinflammatory cytokines. In MCF7 cells, OSM induced a greater than 3.5-fold increase in LOXL2 protein expression, a ~ 2.5-fold increase with IL-1β treatment, but no change with the rest of IL-6-family cytokines (Fig. 2a). MDA-MB-468 cells showed a ~ 2-fold induction of LOXL2 protein expression with OSM treatment, a slight upregulation by IL-1β (not significant), and no change with IL-6 or LIF (Fig. 2b). BT474 cells showed the largest increase in LOXL2 expression with a ~ 10-fold increase, with LIF also promoting a ~ 3-fold induction (Fig. 2c). While with the Sk-Br-3 cells, OSM induced the expression of LOXL2 ~ 2-fold and IL-6 promoted a ~ 3-fold increase in LOXL2 protein (Fig. 2d). None of the cytokines induced a significant change in LOXL2 protein expression in highly invasive MDA-MB-231 cells that constitutively express very high levels of LOXL2 (Fig. 2e), and T47D (ER+ PR+ HER2−) cells did not express any LOXL2 protein before or after treatment (data not shown). The bands represent the 105 kDa LOXL2 protein, which we expect correlates to the secreted and enzymatically active form of LOXL2 that is glycosylated at the N593 and N627 amino acids [50]. LOXL2 protein induction was highest after 24 h, which was supported by In-Cell Western analysis (Supp. Figure 2). These results confirm that OSM signaling, and to a lesser extent IL-1β signaling, induce LOXL2 protein expression in IDC cells, while other IL-6-family cytokines do to a much lesser extent.

Expression of LOXL2 in breast cancer cells is positively correlated with the invasive potential of the breast tumor cells [34, 41]. Therefore, we wanted to determine and compare relative LOXL2 expression between the IDC cell lines treated with OSM. To compare relative expression, immunoblot analysis was performed on lysates from non-treated and OSM-treated cells after 24 h. We observed a significant stepwise increase in constitutive LOXL2 protein expression from the least (MCF7) to most (MDA-MB-231) aggressive cell line. OSM treatment promoted the expression of LOXL2, bridging the gap in LOXL2 expression between the cell lines (Fig. 2f). Our results suggest that OSM-induced LOXL2 protein expression may be correlated to the development of more aggressive invasive ductal carcinomas due to the incremental increase in LOXL2 with OSM exposure. Taken together these results confirm OSM-induced LOXL2 at the mRNA level leads to LOXL2 protein expression, which is correlated with increasing aggressiveness of IDC cells.

### OSM induction of lysyl oxidases is unique to LOXL2

To characterize the effects of OSM signaling on the expression of the different family members of lysyl oxidase, we performed immunoblot assays using MCF7 and MDA-MB-468 cells that were treated for 24 h with OSM. LOX expression was analyzed with IL-6 and LIF treatments, in addition to OSM. Besides LOXL2, the only lysyl oxidase detectable by immunoblot analysis in MCF7 cells was LOXL1. OSM treatment, however, did not alter any of the other lysyl oxidase members (Fig. 2g). These results suggest that OSM induces only LOXL2 expression in the IDC cell lines. Based on these results, we chose to further focus on the OSM-LOXL2 axis in IDCs cells using MCF-7 cells as our model system. Though OSM also exclusively induced the expression of LOXL2 in MDA-MB-468 cells, these cells constitutively expressed LOX protein (Supp. Figure 3). This high endogenous expression of LOX may represent a confounding variable for functional analysis of LOXL2. Taken together, OSM signaling does not impact the expression of all lysyl oxidases but seems to be unique to LOXL2.

### OSM-induced EMT is independent of LOXL2 expression

**Fig. 3:**
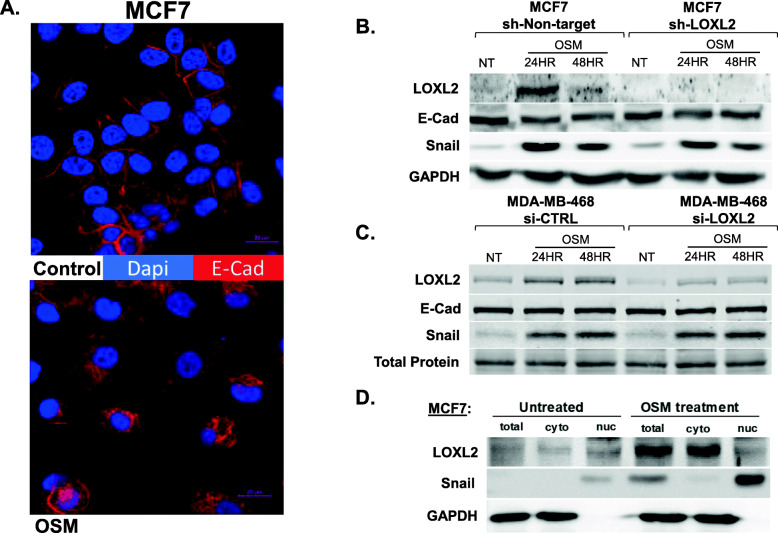
OSM signaling promotes an EMT that is independent of LOXL2 expression. **a** Confocal images of MCF7 cells depict a distinct loss of cell polarity and a transition from membrane localization to cytoplasmic localization of E-cadherin with 48-h OSM treatment, both hallmarks of EMT. E-cadherin (red) and nuclei (DAPI, blue). Magnification × 40 with digital zooming × 2; scale bar = 20 μm. **b** Immunoblot of MCF7-sh-non-target and MCF7-sh-LOXL2 cells treated with OSM for 24 and 48 h. Expression of EMT markers, E-cadherin (E-Cad), and Snail are compared between these cell lines. The absence of LOXL2 expression in MCF-7 cells had no effect on OSM-induced EMT. **c** Immunoblot of MDA-MB-468 breast cancer cells exposed to siCTRL and siLOXL2 for 48 h prior to OSM treatment for 24 and 48 h. Expression of EMT markers, E-Cad, and Snail are compared between siLOXL2 and siCTRL treatments. Inhibited LOXL2 expression in MDA-MB-468 cells had no effect on OSM-induced EMT. **d** Immunoblot of MCF7 cells treated with OSM for 24 h; post treatment cells are collected and subjected to nuclear-cytoplasmic protein fractionation. LOXL2 protein expression is not present in the nuclear fraction, only in the cytoplasmic fraction. GAPDH protein expression is used to confirm purity of cytoplasmic fraction, and Snail transcriptional factor expression is used to confirm nuclear fraction purity. (All experiments *n* = 3+)

EMT has been widely implicated in regulating cell invasion and metastasis [81]. OSM signaling and LOXL2 nuclear localization have been implicated in promoting epithelial to mesenchymal transition in ductal carcinoma cells [17–19, 43, 56, 82]. Indeed, MCF7 cells treated with OSM induced cytoplasmic localization of the epithelial marker E-cadherin (E-Cad) as depicted by immunofluorescence analysis (Fig. 3a). Given that OSM induces LOXL2 expression and the latter has been also implicated in promoting EMT, we next explored whether EMT induced by OSM is dependent on LOXL2 expression. To this end, MCF7 cells stably expressing shRNA targeting LOXL2 (MCF7-sh-LOXL2) and MCF7 cells stably expressing sh-Non-target (MCF7-sh-Non-target) were treated with OSM, and expression of E-cadherin and Snail, a transcription factor mediating EMT, were determined by immunoblot analysis. Our results demonstrate that knockdown of LOXL2 in MCF7 cells did not inhibit OSM-induced EMT, given that E-cadherin expression was slightly downregulated and Snail expression was upregulated upon OSM treatment in both MCF7-sh-Non-target and MCF7-sh-LOXL2 cells (Fig. 3b). Similarly, in MDA-MB-468, our results demonstrate that knockdown of LOXL2 in MDA-MB-468 cells did not inhibit OSM-induced EMT, given that E-cadherin expression did not change and Snail expression was upregulated with OSM treatment in both siCTRL and siLOXL2-exposed MDA-MB-468 cells (Fig. 3c).

Notably, we previously demonstrated that nuclear expression of LOXL2 is required to promote EMT in MCF7 cells [43]. Therefore, we determined the cellular localization of LOXL2 upon OSM induction. We envisioned that OSM induced only cytoplasmic expression of LOXL2, thus promoting EMT independent of LOXL2 expression. To this end, we performed nuclear and cytosolic fractionation on MCF7 cells treated with OSM, using GAPDH as a cytosolic marker and Snail as a nuclear marker (Fig. 3d). Indeed, OSM induced cytoplasmic expression of LOXL2 while the nuclear fraction did not contain any nuclear LOXL2. This data confirmed that OSM did not induce nuclear LOXL2 expression where it could promote EMT through the stabilization of Snail. Taken together, these results demonstrate that OSM promotes EMT independently of its induction of LOXL2 protein.

### OSM induces a glycosylated LOXL2 that is secreted and enzymatically active

**Fig. 4:**
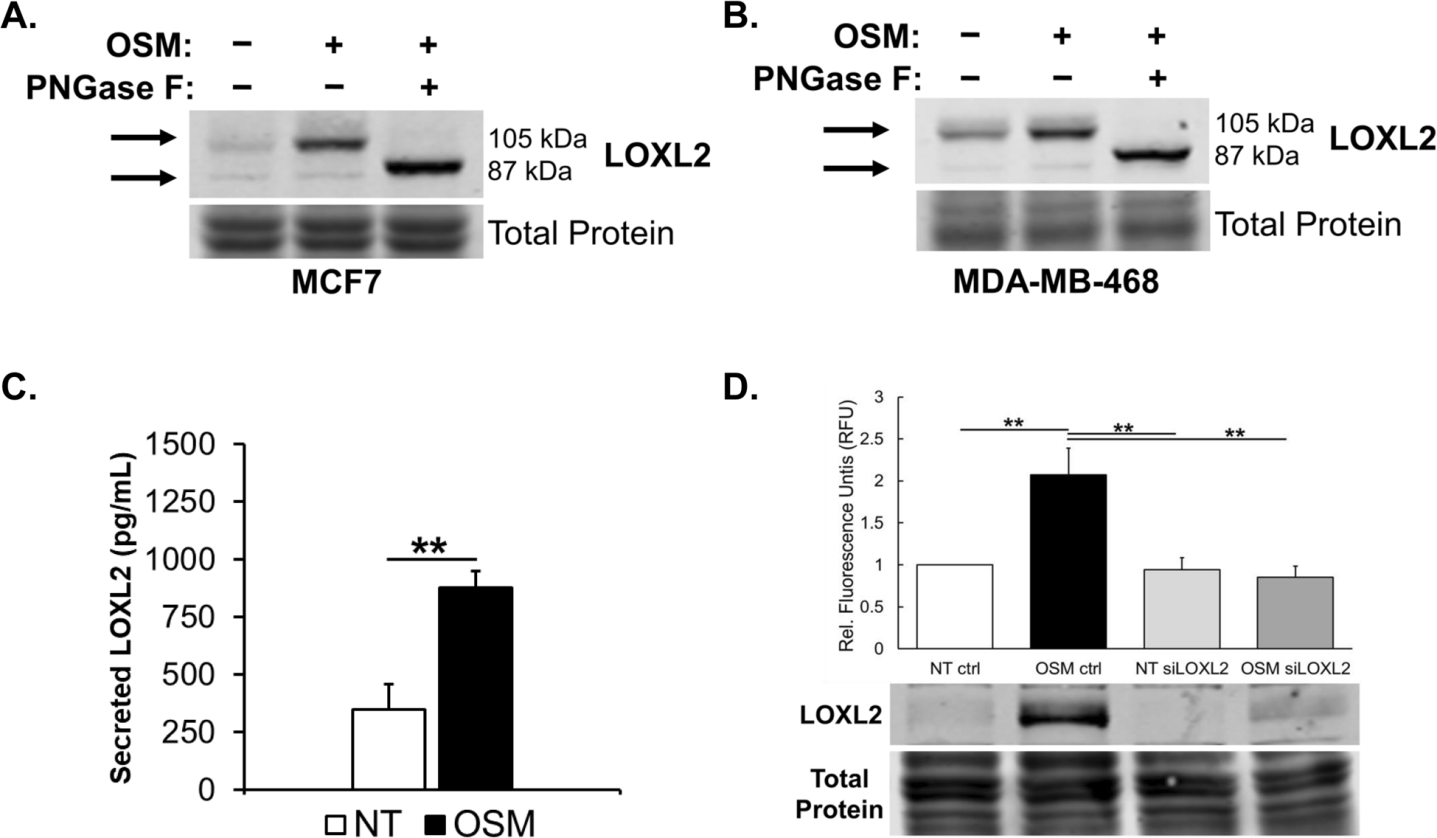
OSM-induced LOXL2 is glycosylated, enzymatically active, and secreted from breast cancer cells. **a** MCF7 and **b** MDA-MB-468 cells were treated with OSM for 24 h to induce the expression of LOXL2. PNGase F, an N-linked glycosylase, is then added to cleave N-linked glycosylation sites. The immunoblot results confirm LOXL2 glycosylation as the LOXL2 protein band size goes from ~ 105 to ~ 87 kDa with PNGase F treatment. **c** Lysyl oxidase activity assay performed on MCF7 cell conditioned media (CM) is analyzed by using an Amplex red-based fluorometric assay. Immunoblot analysis is utilized to confirm siLOXL2 knockdown of LOXL2 expression. Results show that 24-h OSM treatment led to significantly increased lysyl oxidase activity, this is repressed with exposure to siLOXL2. **d** ELISA is used to quantify LOXL2 protein secreted into CM from MCF7 cells after 36 h with OSM treatment. The results confirm that OSM signaling induces the expression, and promotes the secretion, of LOXL2 protein. (All experiments *n* = 3+; *p < 0.05, **p < 0.01, ***p < 0.001; Students *t* test)

LOXL2, in addition to its cell autonomous activity, has been well studied for its extracellular activity on ECM proteins [38, 83]. Secreted LOXL2 promotes collagen I fiber crosslinking and affects matrix remodeling, which has been linked to increased metastatic capability in breast cancer [38–42]. Interestingly, immunoblot analysis suggested that OSM-induced expression of the N-linked glycosylated form of the LOXL2 protein (105 kDa) which is secreted into the tumor microenvironment [50]. To confirm that OSM-induced the expression of glycosylated and enzymatically active LOXL2, we determined the N-linked glycosylation status of expressed LOXL2. MCF7 and MDA-MB-468 cell lysates treated with OSM were exposed to the N-linked deglycosylase enzyme, PNGase F, before immunoblot analysis was performed. OSM induced the expression of the 105 kDa LOXL2 protein, which was reduced in size to 87 kDa following the addition of PNGase F in both MCF7 (Fig. 4a) and MDA-MB-468 (Fig. 4b) cells. To confirm and quantify LOXL2 secretion, we performed an ELISA on MCF7 cells treated with OSM for 36 h. We observed a significant induction in LOXL2 protein secretion with OSM treatment averaging 877 pg/mL of LOXL2 in solution. In comparison to the non-treated samples that averaged 347.6 pg/mL of LOXL2, we observed an ~ 2.5-fold induction in LOXL2 secretion (Fig. 4c). Together, these results confirm that the LOXL2 protein expressed through OSM signaling in IDC cells is N-linked glycosylated and secreted.

In order to confirm that the LOXL2 protein induced by OSM is enzymatically active, we performed a lysyl oxidase activity assay on MCF7 cell conditioned media. MCF7 cells were transfected with siLOXL2 or siCTRL and treated with OSM for 24 h. In the siLOXL2 group, we observed that OSM-treated samples had significantly reduced lysyl oxidase activity compared to the siCTRL group (Fig. 4d). We saw a significant ~ 2-fold increase in lysyl oxidase activity with OSM treatment when comparing against non-treated controls in the siCTRL group. The accompanying immunoblot confirms the knockdown of LOXL2 protein in the MCF7 cell line. Further immunoblot analysis confirmed that there was no impact on LOXL1 protein expression (Supp. Figure 4). Based on these results, we conclude that OSM-induced LOXL2 is enzymatically active and accounts for all of the lysyl oxidase enzymatic activity present in MCF7 cell conditioned media.

### OSM-induced LOXL2 leads to ECM remodeling and increased collagen I fiber alignment

**Fig. 5:**
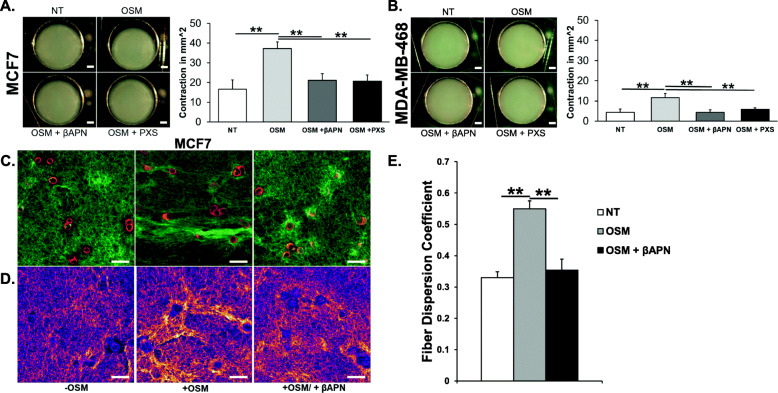
OSM-induced LOXL2 promotes ECM crosslinking and alignment of collagen I fibers. **a** Collagen contraction assay was performed using 1.5 mg/mL collagen I matrices or “pucks” seeded with MCF7 cells and treated for 48 h with OSM or OSM with βAPN (500 μM) or PXS-5120A (200 nM) [LOXL2 inhibitors]. After 48 h, dissecting microscope images of the collagen I matrix depict significantly more contraction in the OSM-treated samples, which is reversed in the presence of LOXL2 inhibitors. Scale bar = 2 mm. Graph quantifying the change in area of the matrix (in mm^2^) due to contraction. Collagen I fiber contraction correlates to fiber crosslinking and is reversed with the inhibition of LOXL2. **b** The same experiment as above was performed with MDA-MB-468 cells. Representative images after 48 h and accompanying graph are depicted using the same scale as above. As with MCF7 cells, LOXL2 inhibition prevented contraction due to OSM-induced LOXL2, but overall contraction is not as pronounced. Scale bar = 2 mm. **c** Confocal images of the collagen “pucks” depict the increase in collagen I fiber (green) alignment between MCF7 cells (red) in collagen I matrices that are treated with OSM for 36 h. Alignment is not observable with the addition of LOXL2 inhibition using βAPN (500 μM). Magnification × 63; scale bar = 50 μm. **d** Representative images with areas of greatest collagen I fiber density emphasized using ImageJ image processing. This clearly highlights the increase in fiber density and alignment present with OSM treatment, which reversed by LOXL2 inhibition. **e** Graph depicting the average fiber dispersion coefficient of collagen I fibers perpendicular to and bridging MCF7 cells in collagen I matrices. Confirms qualitative data that OSM treatment significantly increases alignment which is reversed with LOXL2 inhibition. (All experiments *n* = 3+; **p < 0.01, ***p < 0.001; Students *t* test and one-way ANOVA)

To assess the effect of OSM-induced LOXL2 on crosslinking collagen I, we performed a collagen contraction assay. MCF7 and MDA-MB-468 cells were seeded into a 1.5 mg/mL rat-tail collagen I matrix, and the cells were treated for 48 h with OSM, a combination of OSM and the pan-LOX inhibitor βAPN (500 μM), or a combination of OSM and the LOXL2/3-specific inhibitor PXS-5120A (200 nM). βAPN, or β-aminopropionitrile, is a small molecule inhibitor (SMI) commonly used as a nonspecific inhibitor for lysyl oxidase proteins [64, 83]; PXS-5120A (PXS-S1A) is a LOXL2-specific inhibitor at a range of concentrations in the nanomolar range [84, 85]. OSM induced a ~ 2.5-fold increase in collagen I contraction in MCF7 cells, as compared to the non-treated control, while OSM-induced contraction was blocked by βAPN and PXS-5120A treatment (Fig. 5a). In MDA-MB-468 cells, we saw a similar ~ 2-fold increase; however, the total contraction was substantially reduced compared to MCF7 cells (Fig. 5b). These results demonstrate that OSM-induced LOXL2 increases collagen I contraction, and suggest that OSM promotes crosslinking through induced LOXL2, as collagen I contraction correlates to collagen crosslinking [64, 65].

To visualize collagen alignment, we performed Live-Cell confocal imaging on MCF7 cells treated with OSM and/or βAPN for 36 h in the collagen I matrix described above. Prior to imaging, the MCF7 cells were exposed to CellTracker Red (depicted in red), and the collagen I fibers were visualized by resonance scanning of the matrix (depicted in green). As seen visually, OSM-promoted collagen I alignment and inhibition of LOXL2 with βAPN reduced fiber alignment (Fig. 5c). Images were processed by ImageJ in order to highlight fiber density at its greatest intensity (Fig. 5d). CurveAlign4.0 software [68, 69] was used to quantify the alignment of collagen I fibers in-between, and tangential to, MCF7 cells by analyzing alignment in selected regions of interest (ROI) (Supp. Figure 5). Analysis of the fiber dispersion coefficient in the ROIs using CurveAlign4.0 showed a significant ~ 2-fold increase in fiber alignment with OSM treatment, as the closer the coefficient is to 1 the more alignment is present. The increase in alignment was significantly reversed with the inhibition of LOXL2, using βAPN (Fig. 5e). These results confirm that OSM-induced LOXL2 leads to collagen I fiber alignment in the ECM.

### OSM-induced LOXL2 leads to increased invasion in 3D collagen I matrix

**Fig. 6:**
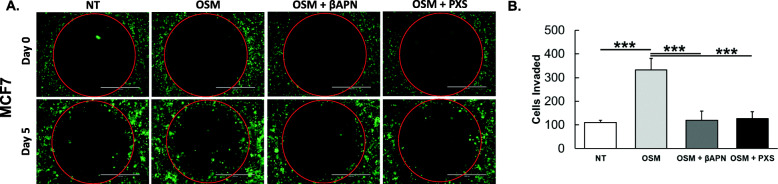
OSM-induced LOXL2 promotes invasion in 3D extracellular matrix. **a** MCF7-GFP cells were seeded into a 1.5 mg/mL collagen I solution and molded into wells of a 96-well plate containing “cell-free zones.” Cells were supplemented with estrogen (50 ng/mL) and treated with OSM, OSM with βAPN (500 μM), or OSM with PXS-5120A (200 nM). Fluorescent images were taken at day 0, and at the conclusion of the experiment on day 5. We observed an increase in 3D invasion of MCF7 cells treated with OSM that was limited by the inhibition of LOXL2 enzymatic function using βAPN or PXS-5120A. Scale bar = 1,000 μm. **b** Graph represents the total number of MCF7-GFP cells that invaded into the cell-free space by day 5, with the various treatments discussed above. OSM treatment significantly increases 3D invasion in MCF7 cells and that is significantly reversed by LOXL2 inhibition. (Experiment *n* = 3; ***p < 0.001; one-way ANOVA)

After determining that OSM-induced LOXL2 promotes a significant increase in collagen I fiber alignment, we wanted to examine whether OSM-induced LOXL2 in turn impacts invasion. Research suggests that an increase in collagen I fiber alignment leads to an increase in invasion of breast cancer cells [52, 53]. To assess the impact of OSM-induced LOXL2 on IDC cell invasive potential, we performed a 3D invasion assay on IDC cells. We utilized MCF7 cells that incorporate green fluorescent protein (GFP) for visualization using fluorescent microscopy. MCF7-GFP cells supplemented with β-estradiol (50 ng/mL) were seeded in a collagen I solution with a concentration of 1.5 mg/mL. The cell suspension was then added to wells of a 96-well plate containing a circular “cell-free zone” for cells to invade towards. Cells were treated with OSM, OSM with βAPN (500 μM), or OSM with PXS-5120A (200 nM) and compared against a non-treated control. Images were taken at day 0, as a control, and the experiment was run until day 5 (Fig. 6a). We analyzed the fluorescent images using ImageJ to determine the number of cells that entered the “cell-free zone” after day 5. The total number of MCF7-GFP cells that entered the “cell-free zone” with OSM treatment increased greater than 3-fold compared to the non-treated control, and cell invasion was in turn significantly decreased by LOXL2 inhibition using both βAPN (~ 3-fold) and PXS-5120A (~ 3-fold) SMIs (Fig. 6b). These results suggest that OSM-induced LOXL2 is critical to OSM-promoted invasion and may likely have impact on metastasis.

Taken together, these results demonstrate that OSM induces sufficient LOXL2 protein expression/secretion to promote remodeling and alignment of collagen I fibers in the ductal carcinoma tumor microenvironment. Due to the alignment of collagen I fibers, it is expected that OSM-induced LOXL2 will promote ductal carcinoma cell invasion and metastasis as tumor cells migrate along aligned collagen fibers [37, 53]. This was supported by our 3D invasion assay results, which showed that OSM induced an ~ 3-fold increase in the number of MCF7-GFP cells that invaded the “cell-free zone,” when compared to OSM treatment in conjunction with LOXL2 inhibition. Therefore, this research highlights a novel mechanism in ductal carcinoma tumor progression, independent of EMT. Further research is needed to confirm that OSM-induced LOXL2 extracellular matrix remodeling leads to a significant increase in metastasis.

## Discussion

**Fig. 7:**
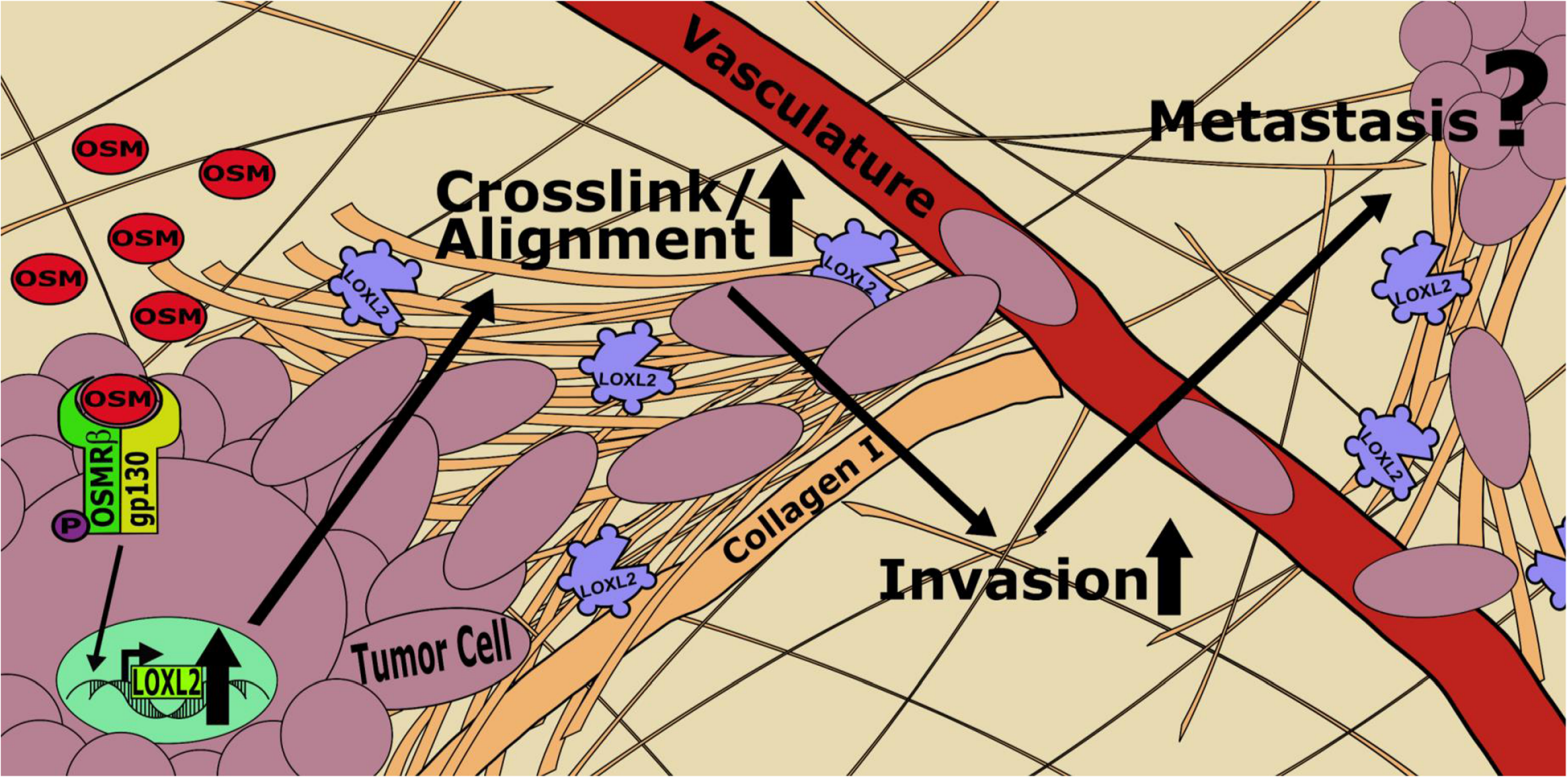
Mechanism by which OSM induces LOXL2 and promotes ECM remodeling. OSM binds to gp130, which recruits OSMRβ to form a heterodimer and allow the phosphorylation and activation of downstream signaling pathways, including STAT3, MAPK, and PI3K. OSM signaling promotes EMT in invasive ductal carcinomas and as the data shows LOXL2 expression in its ~ 105 kDa glycosylated form. The 105 kDa LOXL2 is enzymatically active and secreted into the ECM of the breast tumor microenvironment. In the ECM, LOXL2 promotes crosslinking of the main constituent of the stroma, collagen I, which leads to collagen I fiber alignment. The alignment of collagen I fibers in the stroma provides pathways for cancer cells that have undergone EMT to invade nearby tissue and vasculature [52, 53]. Therefore, these changes to the ECM of the tumor microenvironment likely play a functional role in invasive ductal carcinoma metastasis [27, 37]

The novel findings in our study demonstrate that the proinflammatory cytokine OSM promotes the expression of LOXL2 in breast cancer cells, which significantly impacts collagen I fiber crosslinking, fiber alignment, and invasion (Fig. 7). Clinically, we show that the co-expression of OSM and LOXL2 in patients leads to significantly lower rates of distant metastasis-free survival (DMFS). Our results confirm that proinflammatory cytokine signaling can lead to key alterations in ECM structure through the regulation of LOXL2. These results also suggest that ECM remodeling through OSM-induced LOXL2 may promote metastatic events due to the alignment of collagen I fibers that make up > 80% of the stromal collagen [30]. This is further confirmed by our 3D invasion data, where OSM-induced LOXL2 was key for IDC cell invasion. The novelty of these findings opens the doors for new paradigms related to proinflammatory cytokine-promoted invasion and metastasis.

As it is currently understood in the literature, OSM signaling promotes metastasis by initiating EMT, inducing VEGF expression and angiogenesis, and the secretion of enzymatic proteins that lead to the degradation of the basement membrane surrounding the invasive ductal carcinoma tumor [17–22]. Our research shows that OSM signaling also led to the crosslinking of collagen I fibers, the primary constituent of the stroma that promotes ECM remodeling in the form of increased fiber alignment due to LOXL2 overexpression. Our research additionally shows that OSM-induced LOXL2 is important for cellular invasion in a 3D matrix similar to the stroma. We tested and analyzed six different cell types (MCF7, MDA-MB-468, BT474, Sk-Br-3, MDA-MB-231, and T47D) of which four showed the capability for OSM-induced LOXL2 expression. Several other cytokines also induced LOXL2 expression but in only one or two cell lines. Only in the T47D cell line was LOXL2 neither expressed nor induced. This knowledge is important, as our lab has previously shown that secreted OSM can bind to type I collagen and other ECM fibers and remain active for extended periods of time [86], thus creating an proinflammatory environment around the breast tumor. This proinflammatory TME provides OSM to the tumor cells as they traverse the ECM. OSM increases the tumor cell invasive capability by inducing an EMT response and, as we have just shown for the first time, upregulating LOXL2 expression.

As has been demonstrated for OSM signaling, LOXL2, when it localizes to the nucleus, has been shown to promote EMT [43, 50] through the stabilization and/or upregulation of Snail-1 [50, 55]. Using cytoplasmic and nuclear fractions, we were able to conclude that OSM-induced cytoplasmic expression of LOXL2 and promoted the secretion of LOXL2. LOXL2 KO experiments confirmed that OSM promotes EMT through Snail-1 upregulation and E-cadherin cytoplasmic localization, in a manner independent of LOXL2 expression. Therefore, while OSM-induced LOXL2 does not play a role in promoting EMT, it does actively remodel the ECM of the TME by promoting collagen I fiber alignment and IDC cell invasion. As previously published, aligned collagen fibers facilitate directed tumor cell migration towards nearby vasculature, an important early step in metastasis [37, 53]. Thus, OSM-induced LOXL2 has the potential to promote higher rates of metastasis, in addition to OSM-promoted EMT. It was recently documented that knockdown of LOXL2 expression in specific lung adenocarcinoma cell lines decreased collagen fibrillar alignment [87]. The presence of LOXL2 in the ECM was also observed to lead to the formation of stiffer matrices [48, 49]. Stiffer substrates provide metastasizing tumor cells better focal adhesion anchorage and “durotaxis,” which leads to easier and faster migration [88, 89]. The ECM remodeling draws a parallel with research that highlights patients with stiff, dense breast tissue have a worse prognosis than those with normal density [90–92].

Our analysis of patient data confirmed that IDC patients with high co-expression of OSM and LOXL2 have worse rates of metastasis than either alone. LOXL2-promoted collagen I fiber alignment in addition to OSM-promoted EMT may be responsible for the drastic decrease in DMFS in IDC patients. This data is supported by published research demonstrating that, individually, OSM and LOXL2 overexpression in patients correlates with decreased reoccurrence-free survival (RFS) and distant metastasis-free survival (DMFS) [38, 41, 70, 71]. Our patient data is further supported by our in vitro data. LOXL2 expression correlates with IDC cell aggressiveness, as the more aggressive MDA-MB-231 and MDA-MB-468 triple negative breast cancer cell lines show higher LOXL2 expression than less aggressive ER+/PR+ MCF7 cells. This phenomenon has been confirmed independently by other labs [34, 38–41]. These results suggest that LOXL2 regulation is critical for OSM signaling promoted invasiveness and metastasis in IDC. These results are important, because previous research has shown that OSM promotes metastatic events through EMT, angiogenesis, and basement membrane degradation. Our research in addition suggests that OSM-induced LOXL2 also promotes metastatic events through the alignment of collagen I fibers found abundantly in the stroma, allowing mesenchymal-like tumor cells to efficiently migrate into vasculature and nearby tissue.

## Conclusion

In summary, we show for the first time that a proinflammatory cytokine (OSM) can promote the expression of ECM remodeling lysyl oxidases, specifically LOXL2, in IDC cells that leads to significant collagen I fiber crosslinking, alignment, and IDC invasion. Because collagen I fiber alignment is associated with increased tumor cell motility rate, and we observed an increase in 3D invasion within a collagen I matrix; OSM-induced LOXL2 may likely have an impact on metastasis. For our future goals, we will perform in vivo studies to determine how OSM-induced LOXL2 affects metastasis and in vitro experiments to determine the transcription factor and signaling mechanism responsible for the induction of LOXL2 by OSM. There is a major need for novel ways to treat and prevent breast cancer metastasis, and the mechanism behind OSM’s induction of LOXL2 could prove to be exploitable in the race for more effective cancer therapies. In addition, OSM expression and signaling is linked to invasion and metastasis in other carcinomas including prostate, cervical, ovarian, kidney, and lung [93–97]. Combined with our correlation data between OSMR and LOXL2 mRNA in glioblastoma, prostate, and ovarian cancer patients, it is possible that OSM induces LOXL2 in multiple types of cancer and these patients could also benefit from a therapeutic targeting OSM induction of LOXL2.

## Supplementary Information

**Additional file 1.**

## Data Availability

The datasets used and/or analyzed during the current study are available from the corresponding author on reasonable request and are freely available online.

## Abbreviations

βAPN: Beta-aminopropionitrile
BME: Basement membrane
CI: Confidence interval
CSC: Circulating tumor cell
DCIS: Ductal carcinoma in situ
DMFS: Distant metastasis-free survival
EMT: Epithelial-to-mesenchymal transition
ECM: Extracellular matrix
IDC: Invasive ductal carcinoma
IL-1β: Interleukin-1beta
IL-6: Interleukin-6
LIF: Leukemia inhibitory factor
LOX: Lysyl oxidase
LOXL2: Lysyl oxidase like-2
LTQ: Lysyl tyrosylquinone
OSMR: Oncostatin M receptor
OSM: Oncostatin M
RFS: Recurrence-free survival
ROI: Region of interest
SMI: Small molecule inhibitor
TCGA: The Cancer Genome Atlas
TME: Tumor microenvironment
VEGF: Vascular endothelial growth factor
