# Supplementary material for: Novel mechanism for OSM-promoted extracellular matrix remodeling in breast cancer: LOXL2 upregulation and subsequent ECM alignment
Source: Breast Cancer Res. 2021 May 19;23:56. doi: 10.1186/s13058-021-01430-x (PMC8132418; doi:10.1186/s13058-021-01430-x)
Supplement: Supplementary file 1 — Additional file 1. [file 13058_2021_1430_MOESM1_ESM.pptx]

## Slide 1
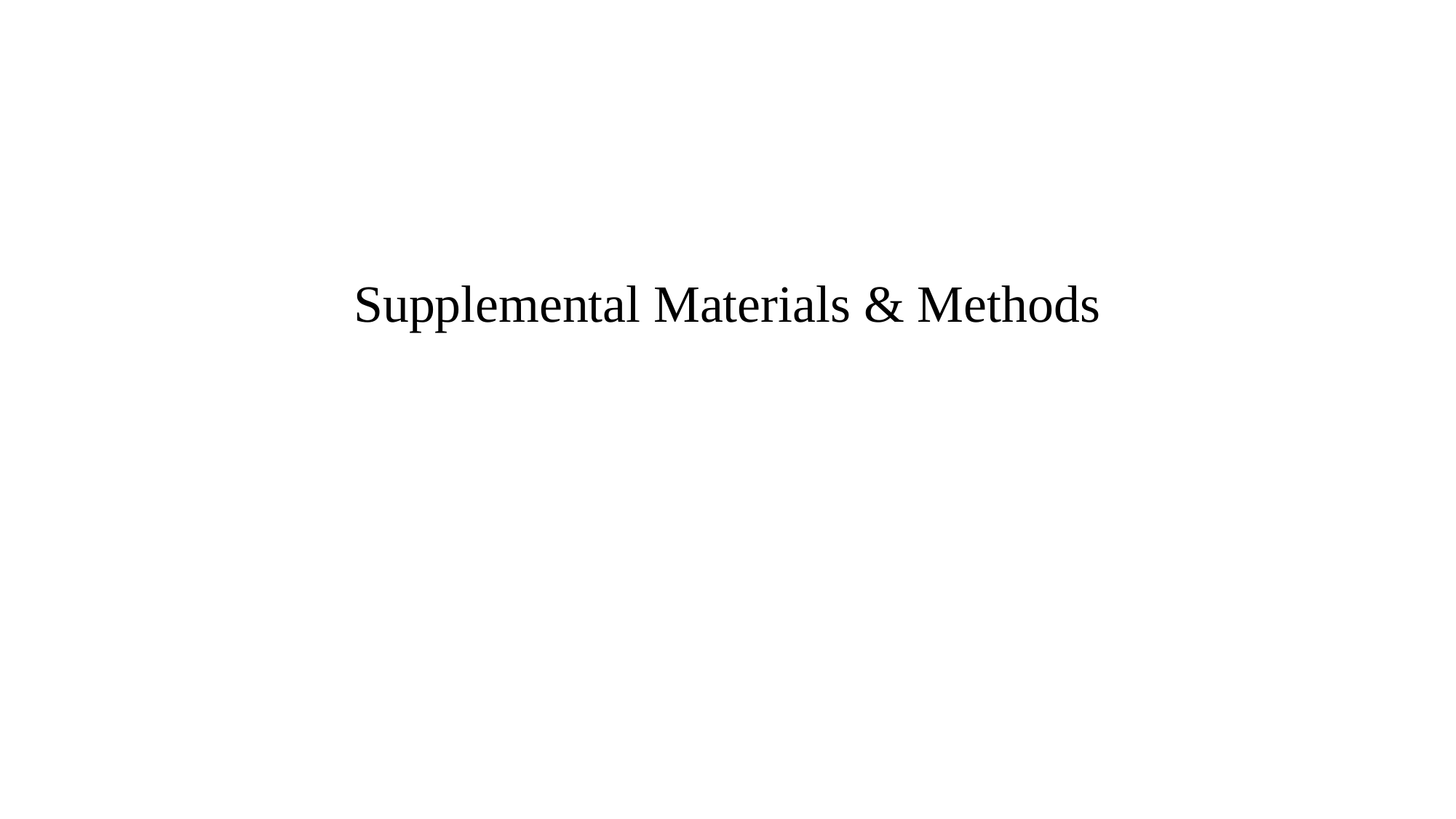

Supplemental Materials & Methods

## Slide 2
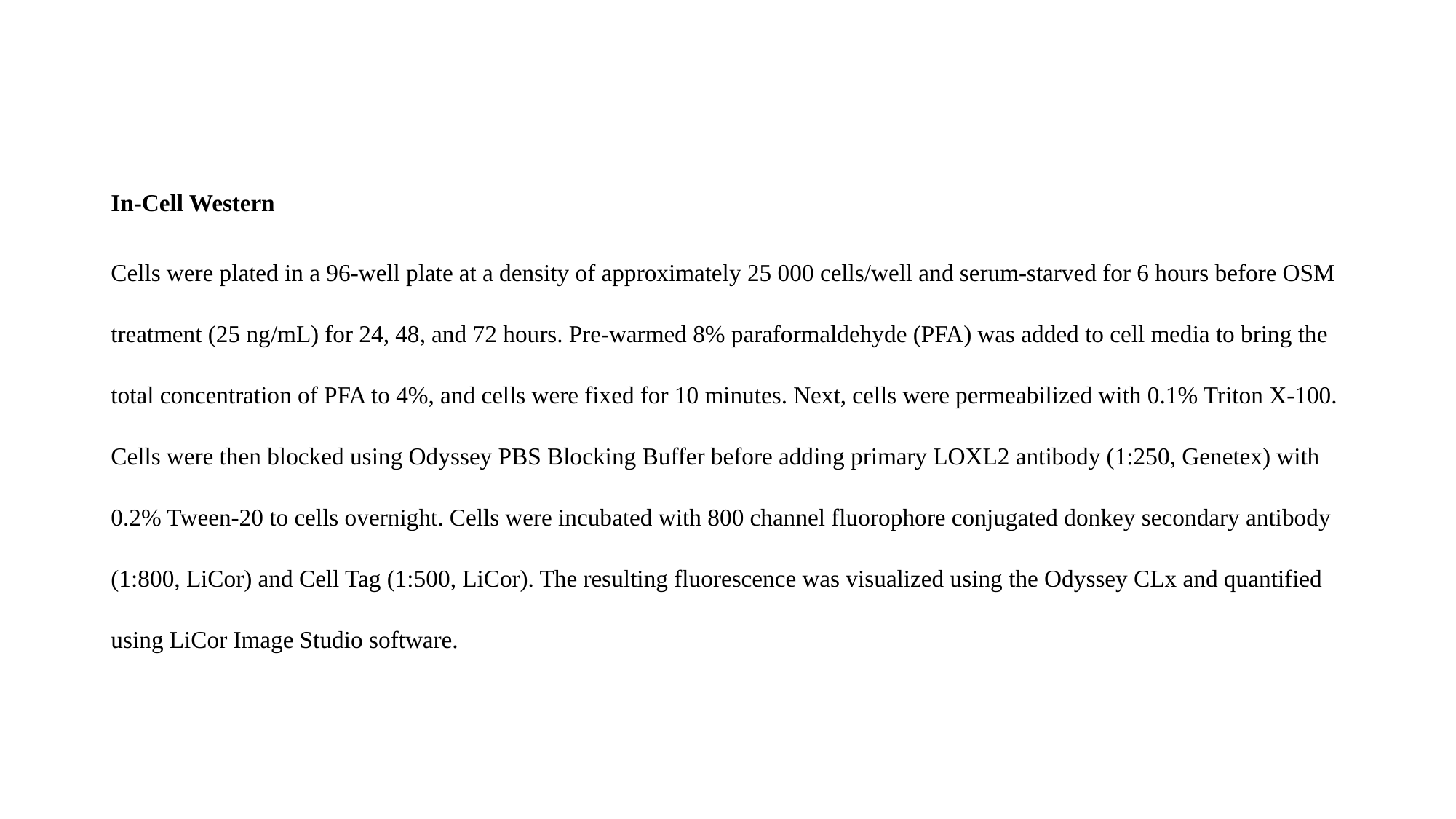

In-Cell Western
Cells were plated in a 96-well plate at a density of approximately 25 000 cells/well and serum-starved for 6 hours before OSM treatment (25 ng/mL) for 24, 48, and 72 hours. Pre-warmed 8% paraformaldehyde (PFA) was added to cell media to bring the total concentration of PFA to 4%, and cells were fixed for 10 minutes. Next, cells were permeabilized with 0.1% Triton X-100. Cells were then blocked using Odyssey PBS Blocking Buffer before adding primary LOXL2 antibody (1:250, Genetex) with 0.2% Tween-20 to cells overnight. Cells were incubated with 800 channel fluorophore conjugated donkey secondary antibody (1:800, LiCor) and Cell Tag (1:500, LiCor). The resulting fluorescence was visualized using the Odyssey CLx and quantified using LiCor Image Studio software.

## Slide 3
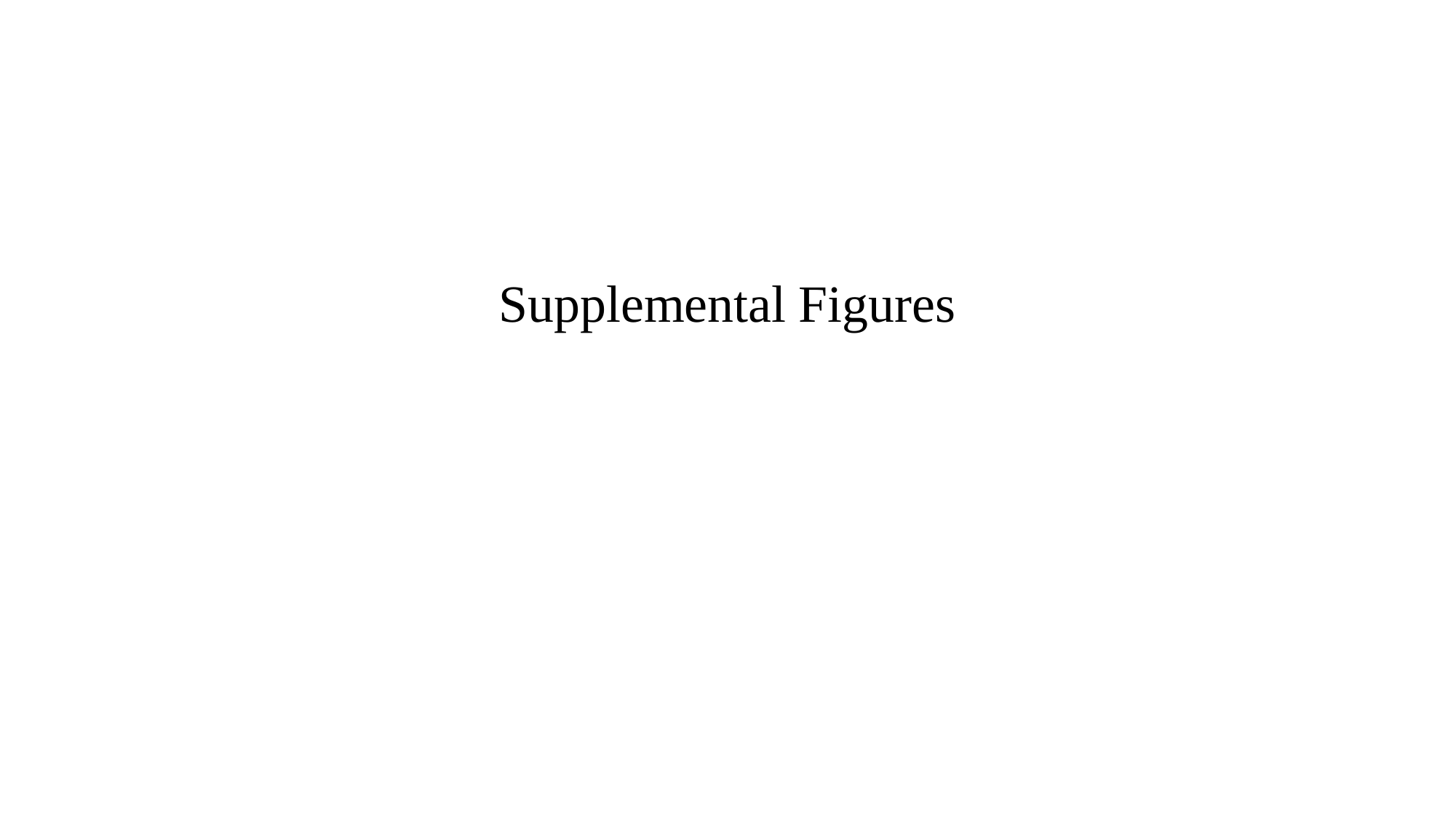

Supplemental Figures

## Slide 4
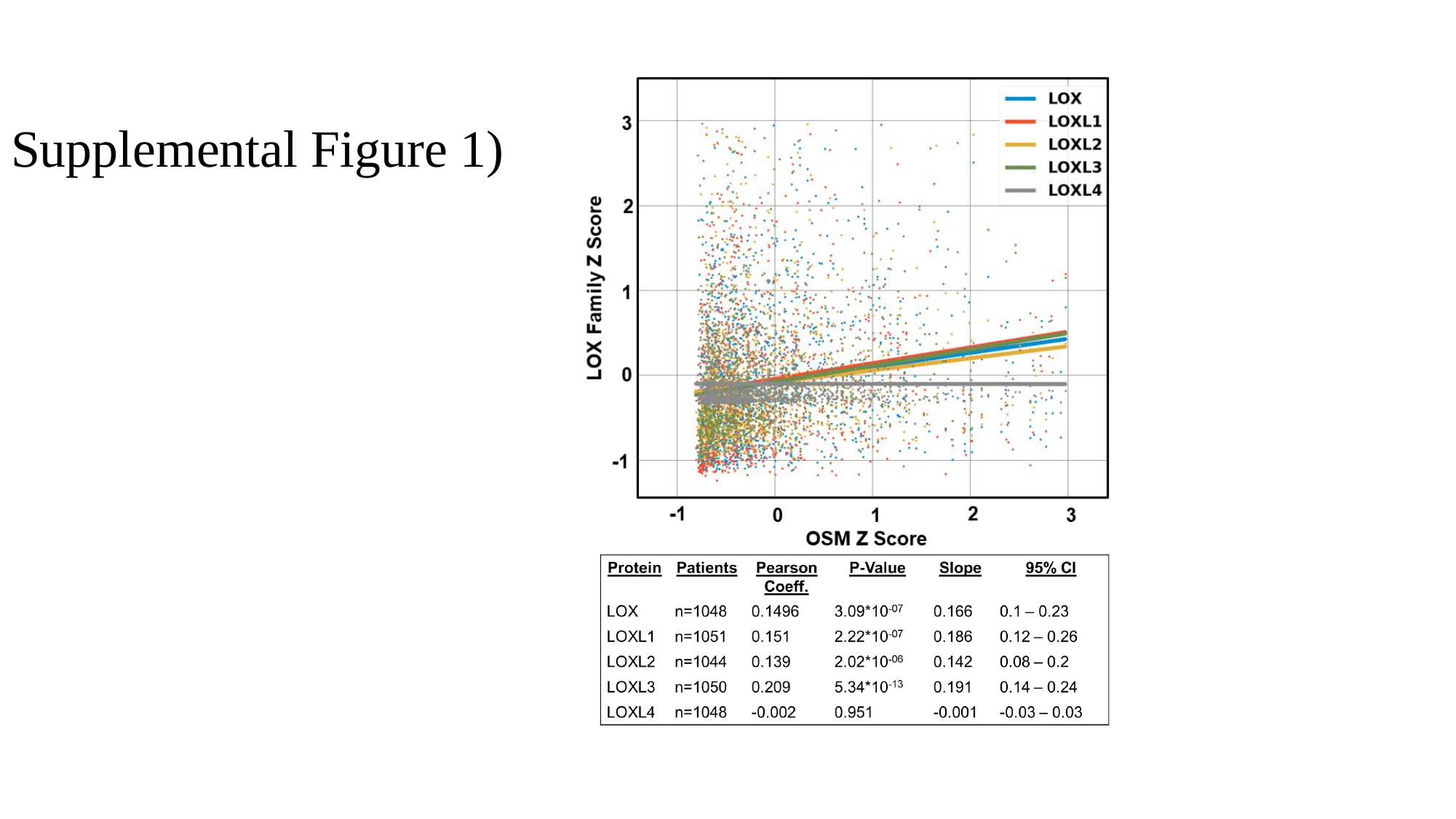

# Supplemental Figure 1)

## Slide 5
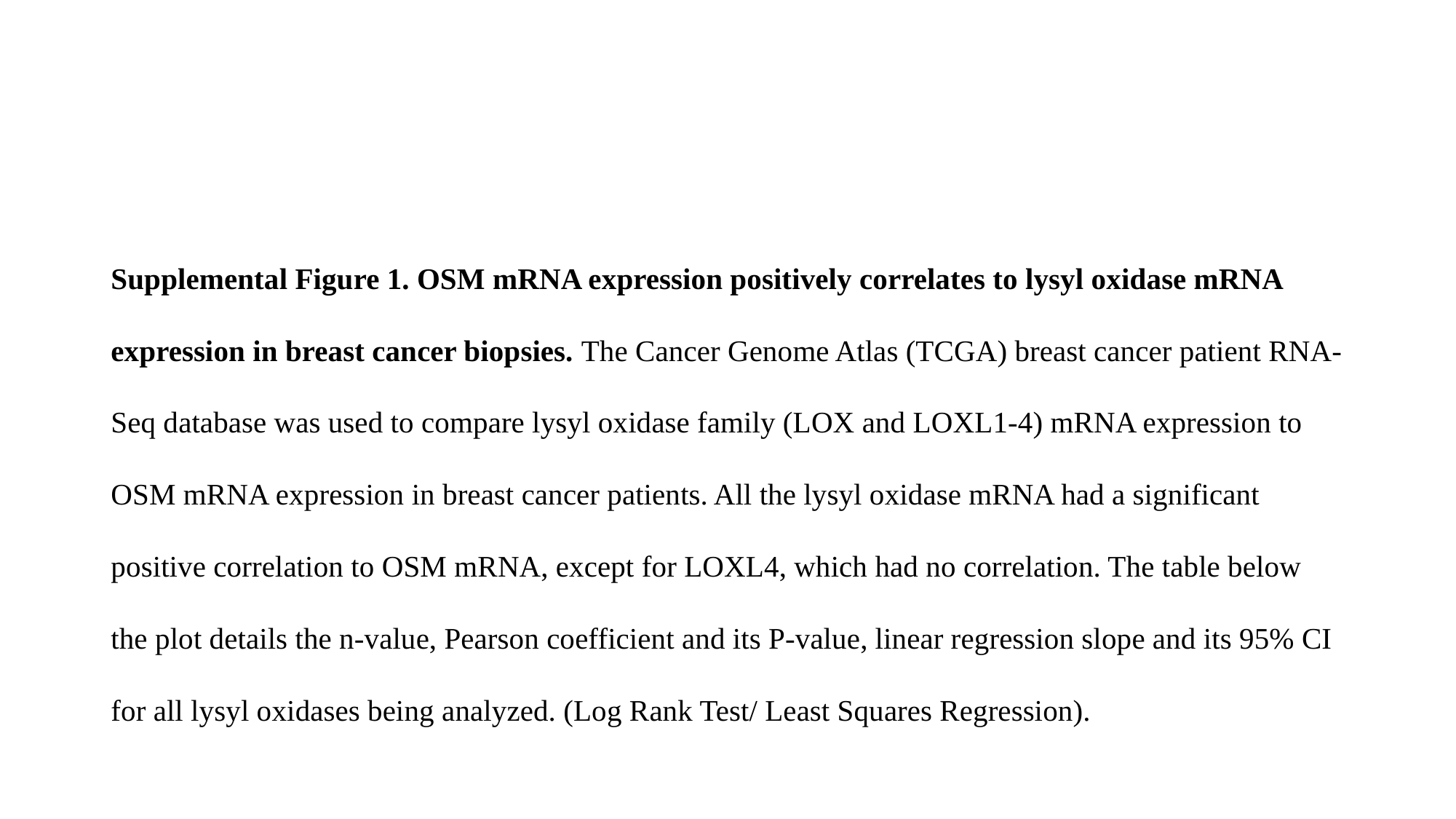

Supplemental Figure 1. OSM mRNA expression positively correlates to lysyl oxidase mRNA expression in breast cancer biopsies. The Cancer Genome Atlas (TCGA) breast cancer patient RNA-Seq database was used to compare lysyl oxidase family (LOX and LOXL1-4) mRNA expression to OSM mRNA expression in breast cancer patients. All the lysyl oxidase mRNA had a significant positive correlation to OSM mRNA, except for LOXL4, which had no correlation. The table below the plot details the n-value, Pearson coefficient and its P-value, linear regression slope and its 95% CI for all lysyl oxidases being analyzed. (Log Rank Test/ Least Squares Regression).

## Slide 6
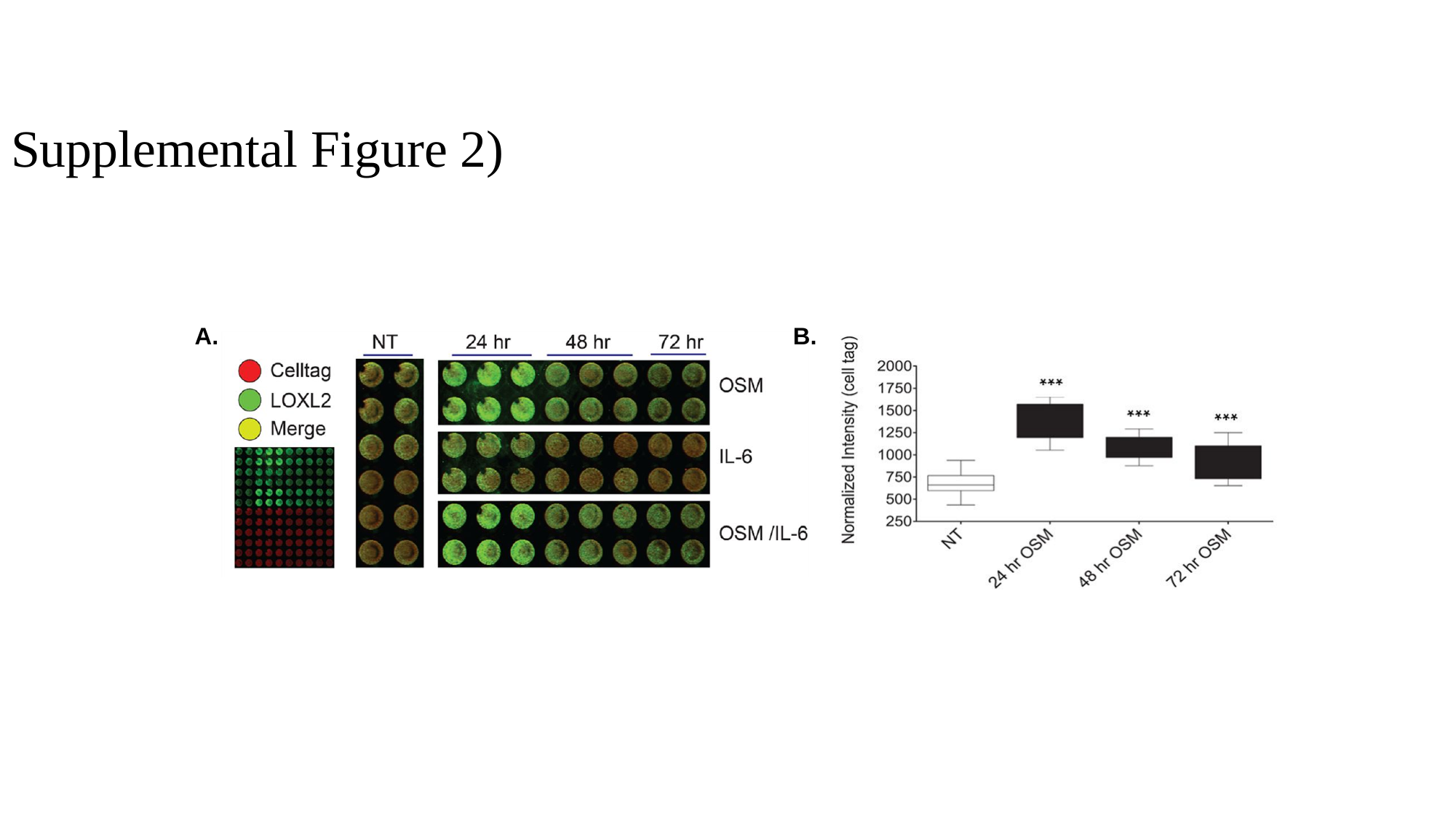

# Supplemental Figure 2)
A.
B.

## Slide 7
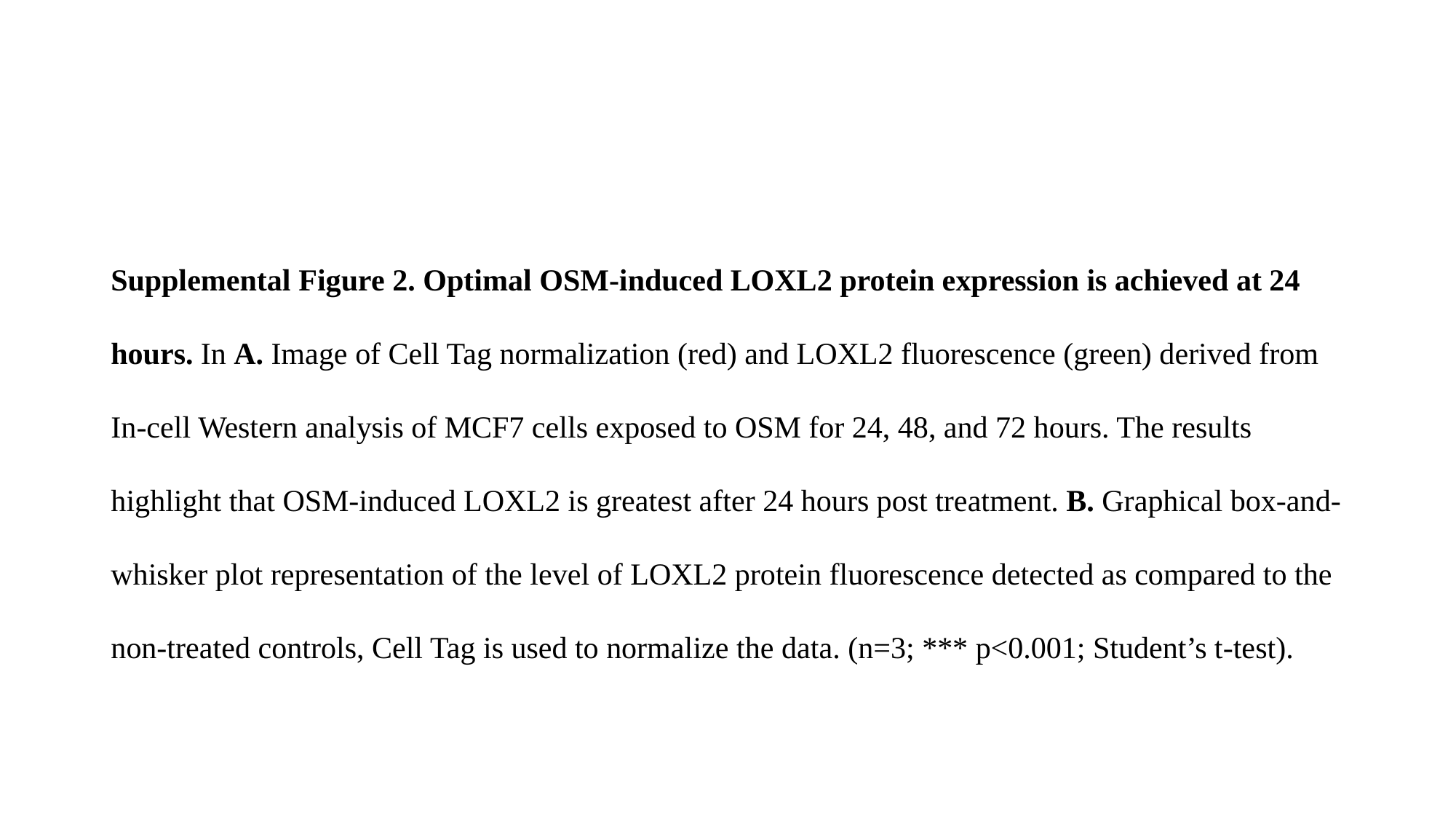

Supplemental Figure 2. Optimal OSM-induced LOXL2 protein expression is achieved at 24 hours. In A. Image of Cell Tag normalization (red) and LOXL2 fluorescence (green) derived from In-cell Western analysis of MCF7 cells exposed to OSM for 24, 48, and 72 hours. The results highlight that OSM-induced LOXL2 is greatest after 24 hours post treatment. B. Graphical box-and-whisker plot representation of the level of LOXL2 protein fluorescence detected as compared to the non-treated controls, Cell Tag is used to normalize the data. (n=3; *** p<0.001; Student’s t-test).

## Slide 8
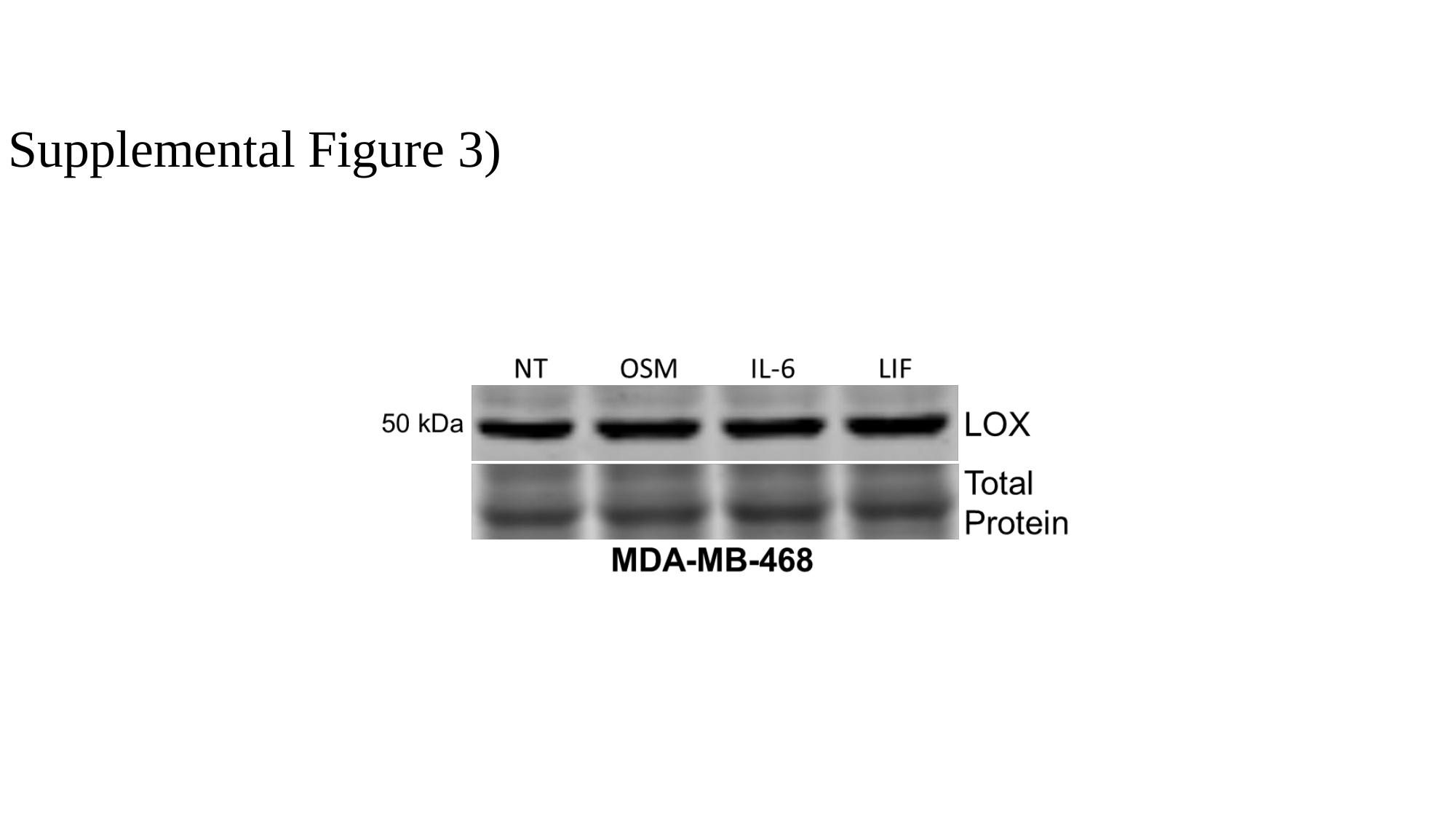

Supplemental Figure 3)

## Slide 9
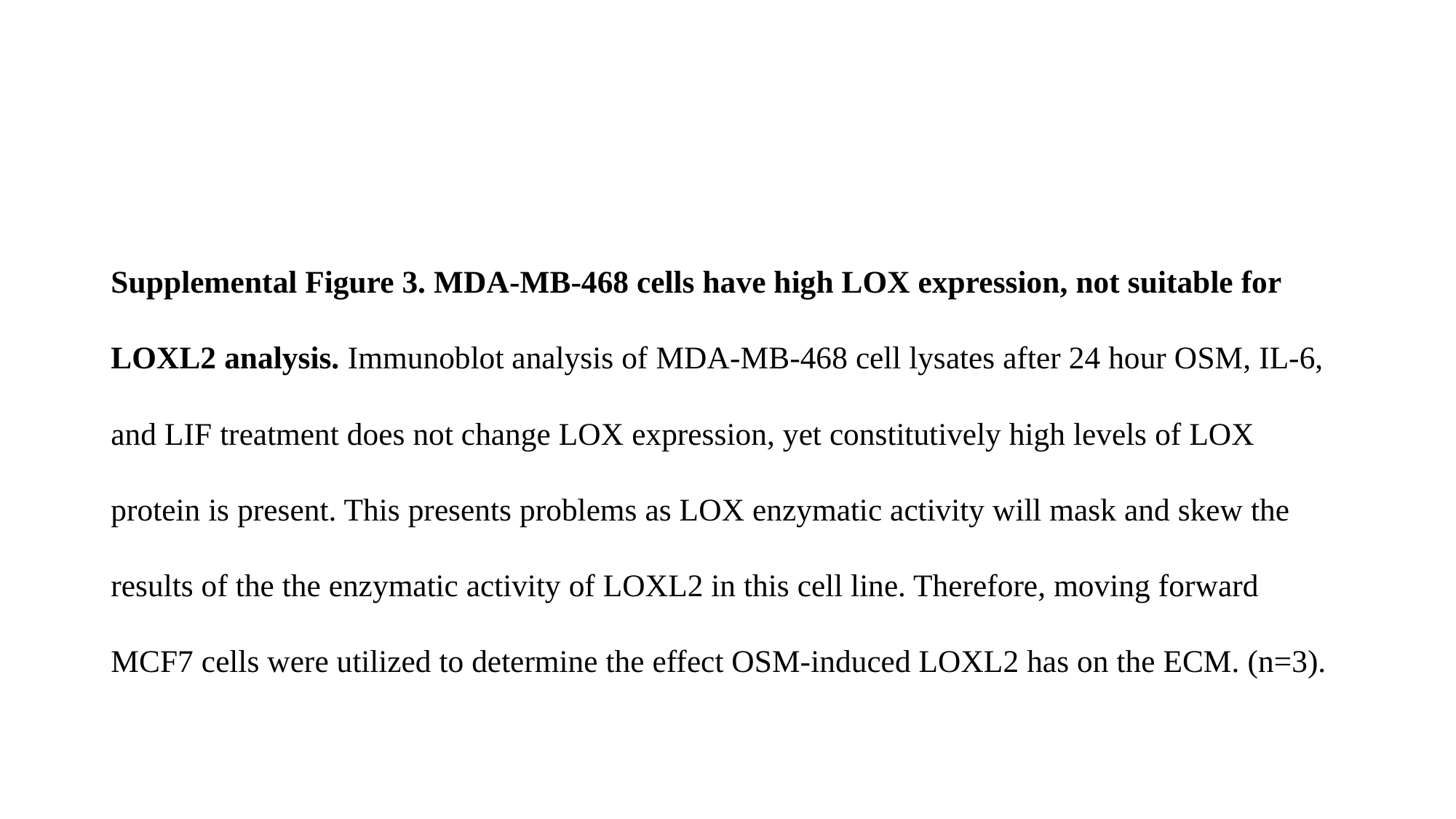

Supplemental Figure 3. MDA-MB-468 cells have high LOX expression, not suitable for LOXL2 analysis. Immunoblot analysis of MDA-MB-468 cell lysates after 24 hour OSM, IL-6, and LIF treatment does not change LOX expression, yet constitutively high levels of LOX protein is present. This presents problems as LOX enzymatic activity will mask and skew the results of the the enzymatic activity of LOXL2 in this cell line. Therefore, moving forward MCF7 cells were utilized to determine the effect OSM-induced LOXL2 has on the ECM. (n=3).

## Slide 10
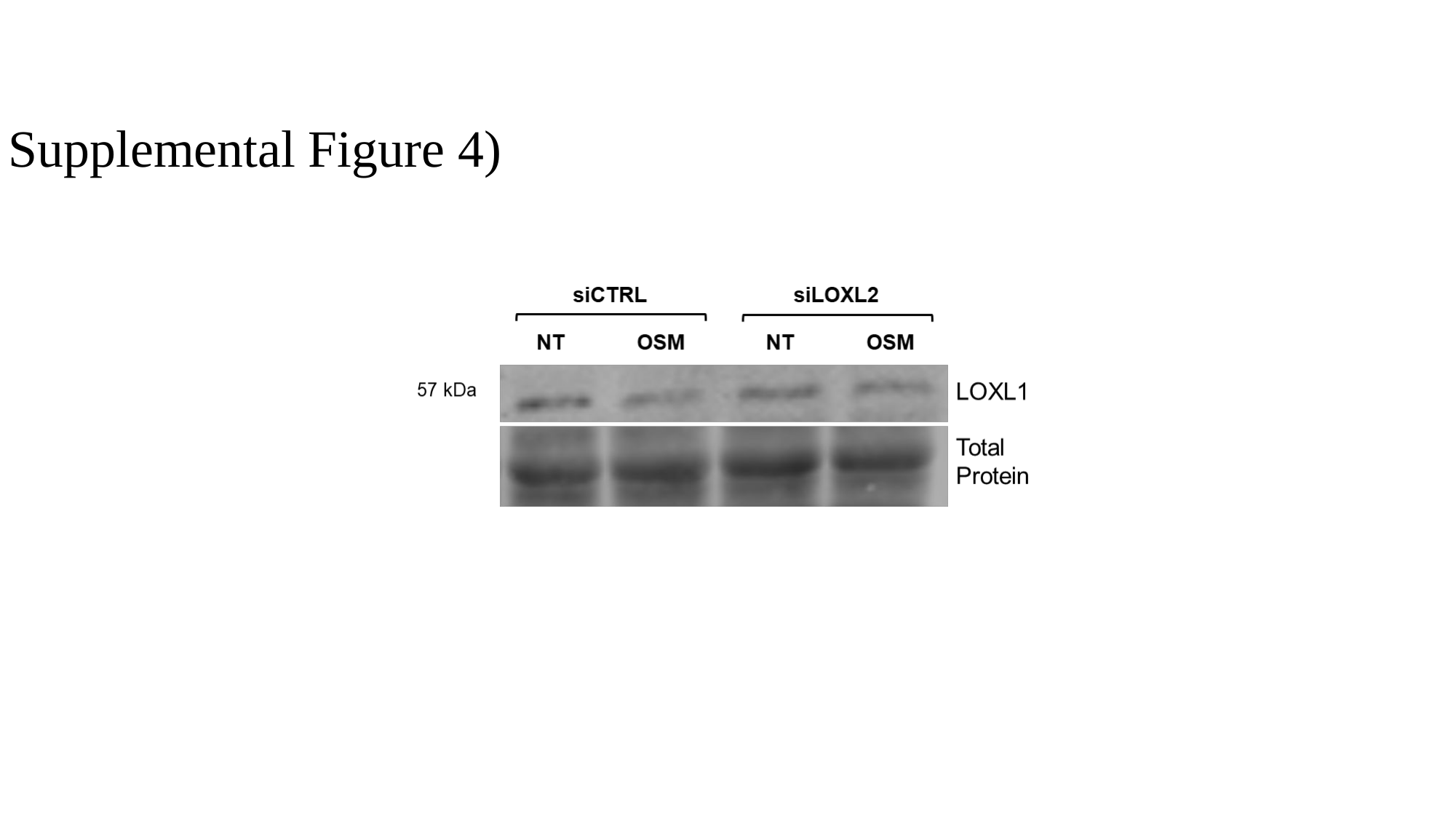

Supplemental Figure 4)

## Slide 11
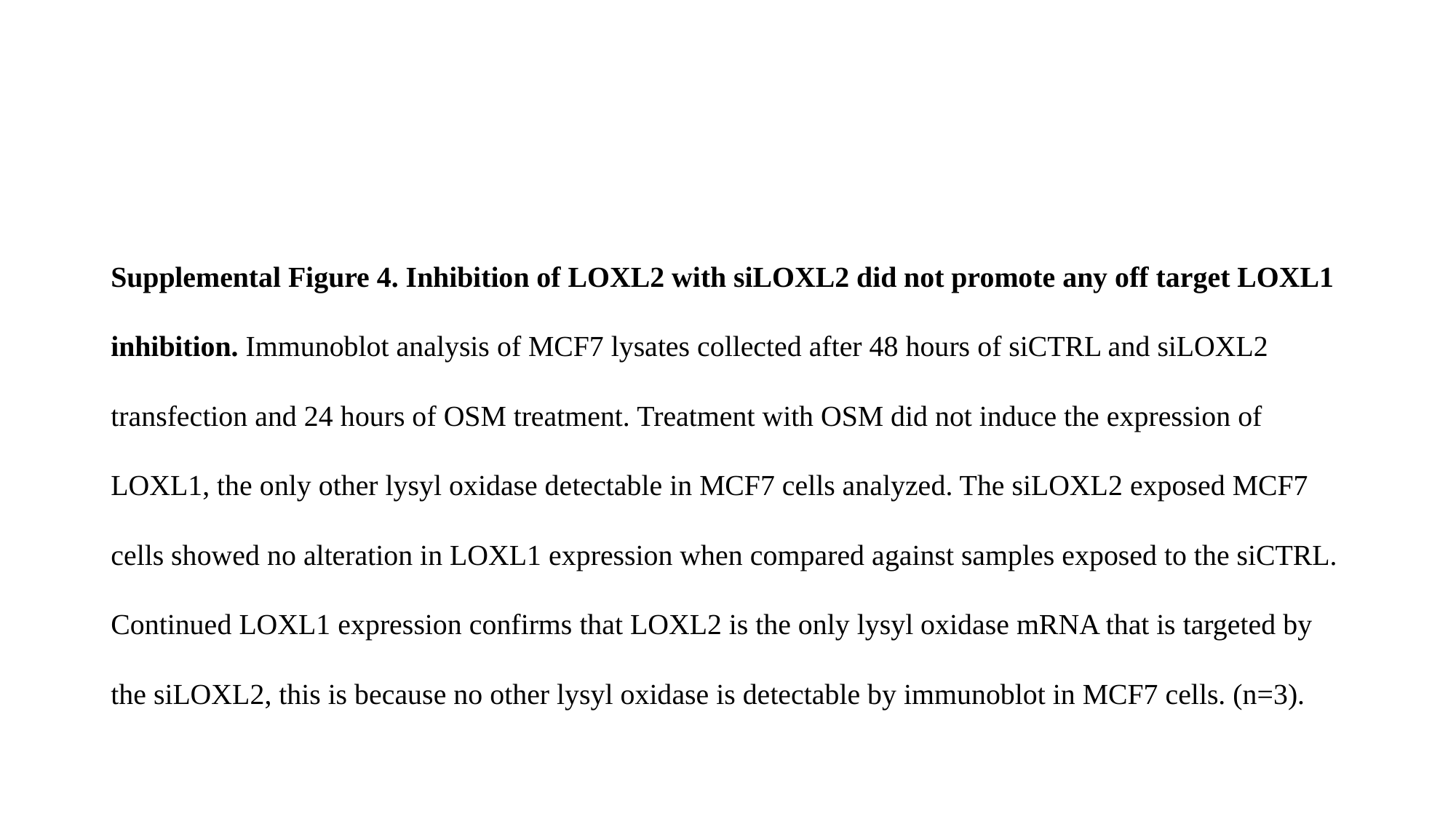

Supplemental Figure 4. Inhibition of LOXL2 with siLOXL2 did not promote any off target LOXL1 inhibition. Immunoblot analysis of MCF7 lysates collected after 48 hours of siCTRL and siLOXL2 transfection and 24 hours of OSM treatment. Treatment with OSM did not induce the expression of LOXL1, the only other lysyl oxidase detectable in MCF7 cells analyzed. The siLOXL2 exposed MCF7 cells showed no alteration in LOXL1 expression when compared against samples exposed to the siCTRL. Continued LOXL1 expression confirms that LOXL2 is the only lysyl oxidase mRNA that is targeted by the siLOXL2, this is because no other lysyl oxidase is detectable by immunoblot in MCF7 cells. (n=3).

## Slide 12
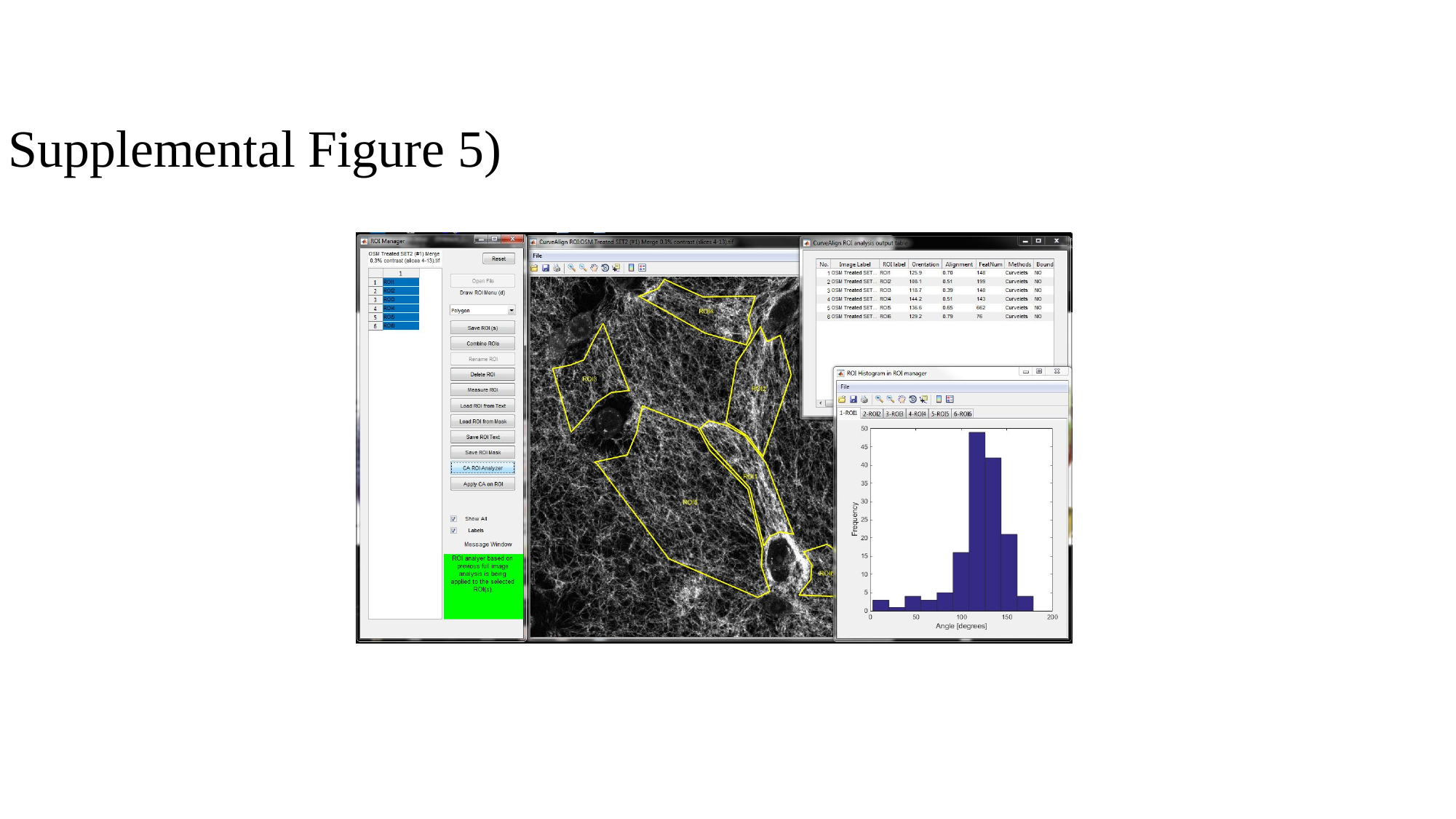

Supplemental Figure 5)

## Slide 13
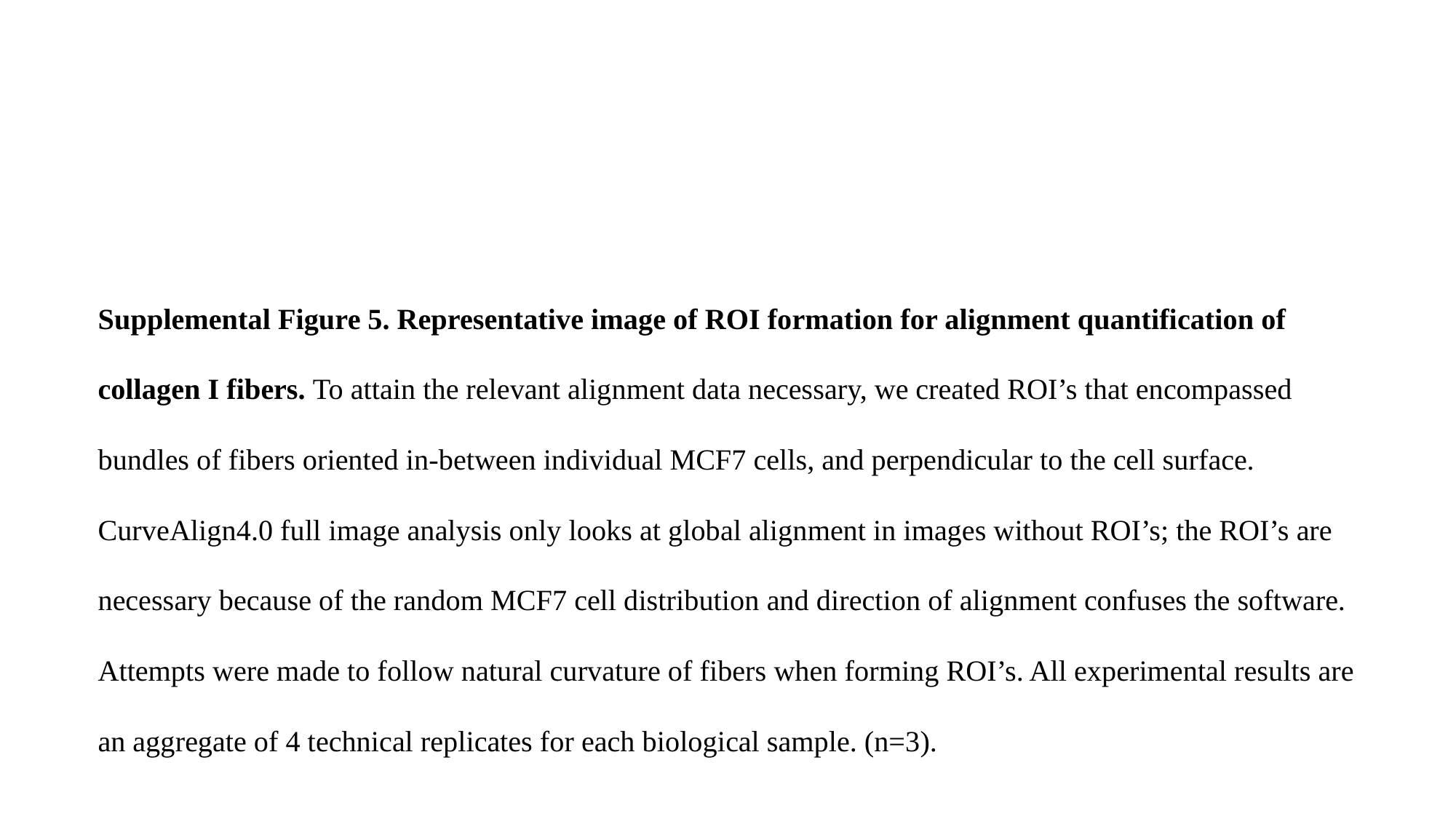

Supplemental Figure 5. Representative image of ROI formation for alignment quantification of collagen I fibers. To attain the relevant alignment data necessary, we created ROI’s that encompassed bundles of fibers oriented in-between individual MCF7 cells, and perpendicular to the cell surface. CurveAlign4.0 full image analysis only looks at global alignment in images without ROI’s; the ROI’s are necessary because of the random MCF7 cell distribution and direction of alignment confuses the software. Attempts were made to follow natural curvature of fibers when forming ROI’s. All experimental results are an aggregate of 4 technical replicates for each biological sample. (n=3).
